# Relationship Between Gaming Disorder, Self-Compensation Motivation, Game Flow, Time Spent Gaming, and Fear of Missing Out Among a Sample of Chinese University Students: A Network Analysis

**DOI:** 10.3389/fpsyt.2021.761519

**Published:** 2021-11-01

**Authors:** Li Li, Zhimin Niu, Mark D. Griffiths, Songli Mei

**Affiliations:** ^1^School of Humanities and Social Sciences, Gannan Medical University, Ganzhou, China; ^2^International Gaming Research Unit, Psychology Department, Nottingham Trent University, Nottingham, United Kingdom; ^3^School of Public Health, Jilin University, Changchun, China

**Keywords:** gaming disorder, self-compensation motivation, game flow, time spent gaming, fear of missing out, network analysis

## Abstract

**Background and Aims:** In previous correlational research, the relationship between gaming disorder (GD), compensation motivation, game flow, time spent gaming, and fear of missing out (FoMO) has been examined. However, network analysis has rarely been applied to explore the relationship between GD, self-compensation motivation, game flow, time spent gaming, and FoMO. Therefore, the present study used network analysis to examine the relationship between the aforementioned variables among a sample of gamers.

**Methods:** The present study comprised gamers (*N* = 1,635) recruited from three Chinese universities, who completed an online survey including the Gaming Disorder Test, Self-Compensation Motivation Questionnaire, Game Flow Questionnaire, and Trait-State Fear of Missing Out Scale, as well as four items related to time spent gaming.

**Results:** Self-compensation motivation, game flow, time spent gaming, and FoMO were all significantly and positively associated with GD. In the domain-level and facet-level networks, weekday gaming hours and weekend gaming hours had the strongest edge intensity. The domain-level, facet-level, and item-level networks analysis also showed that GD was connected with self-compensation motivation, game flow, time spent gaming, and FoMO. The network structure demonstrated a significant difference between males and females (2.33 vs. 2.81, *p* = 0.001) using the domain-level network comparison test (NCT).

**Conclusions:** The results suggest that GD is closely associated with self-compensation motivation, game flow, time spent gaming, and FoMO. FoMO and gaming motivation (i.e., self-compensation and game flow) may increase time spent gaming and facilitate GD. Therefore, interventions that decrease game immersion and time spent gaming are likely to decrease GD.

## Introduction

### Gaming Disorder

The diagnosis of gaming disorder (GD) in the eleventh revision of the International Classification of Diseases (ICD-11) includes three core symptoms: (i) impaired control over gaming, (ii) increasing priority to gaming more than other life interests and daily activities, and (iii) continuation or escalation of gaming despite negative outcomes (1). In addition, GD will lead to marked distress and significant impairment in personal, family, social, educational, occupational, or other important aspects of functioning (1). In China (where the present study was carried out), the prevalence of internet gaming disorder (IGD) among young gamers (aged 15–25 years) was reported to be 17% (2, 3), which was similar to previous research (4). In addition, according to the expert consensus on the prevention and treatment of gaming disorder in China, the prevalence of GD is ~5% among the total population (5).

The Interaction of Person-Affect-Execution (I-PACE) model proposes that addictive behaviors (e.g., gaming disorder and gambling disorder) develop as a result of the interactions between predisposing variables [i.e., general (e.g., genetics and temperamental features) and behavioral-specific (e.g., specific need and motives)], affective and cognitive responses to triggers, and executive functions (e.g., inhibitory control and decision-making) (6). Personality traits and emotion dysregulation as potential vulnerability factors for problematic internet use (PIU) have been shown to be associated in studies of among American young adult (7), while the association between impulsivity with PIU has been found among young Italians (8). Gaming disorder has also been considered as a consequence of deficient self-regulation and need gratification based on the dilution effect hypothesis (9). Moreover, self-determination theory posits that IGD is associated with daily frustration of basic needs (i.e., relatedness, autonomy, and competence) and stronger extrinsic gaming motivations (10). It has also been found that the Dark Triad of personality traits (i.e., narcissism, Machiavellianism, and psychopathy) can influence gaming disorder via gaming motivations (e.g., socializing, escapism, and achievement) (11, 12). Need-fulfillment deficits have also been associated with IGD (13). In addition, variables such as time spent gaming and anxiety may predict IGD symptoms (14). Other personality traits (e.g., neuroticism and impulsivity) (15–18), as well as state anxiety and trait anxiety also may predict GD (19).

### Self-Compensation Motivation

Self-compensation occurs when individuals take action to compensate for threats and discomfort originating from socio-psychological stress (20). For some individuals, internet use behavior can be regarded as a compensation when the process of adolescents' mental development is blocked (e.g., self-identity crisis or maladaptive interpersonal relationships), and may result in IGD due to problematic compensation (i.e., excessive internet use) (21). This has been referred to as the psychological decompensation hypothesis which posits that: (i) the smooth state of individual development is normal development; (ii) the state of blocking development may occur due to the interaction of external and internal causes; (iii) at the stage of blocking development, normal development may be recovered through constructive compensation which activates the process of psychological self-repair; and (iv) decompensation indicates that lack of psychological self-repair may lead to deviation or interruption of development through problematic compensation (21). In addition, according to the model of compensatory internet use, some individuals go online to alleviate negative emotion or escape real life problems as a response to coping with negative life events, which may cause self-compensation and further lead to problematic internet use when using internet excessively (22).

Some research has noted that individuals with higher social anxiety may have stronger self-compensation motivation due to more insatiable social need. Online gaming has been viewed as a means or tool of self-compensation which may relieve anxiety, provide achievement, foster social affiliation and/or help escape real life issues (23–26). Gaming motivation, especially escapism, has been shown to mediate the relationship between psychiatric distress and gaming disorder among both esport gamers and recreational gamers (27). Gaming motivation (e.g., escapism, achievement, and socializing) has also been reported as one of risk factors in the development of GD (26). Moreover, a recent study reported that self-compensation motivation mediated the relationship between perceived stress and IGD, and that gender moderated the relationship between perceived stress and self-compensation motivation (28).

### Game Flow

Flow refers to the optimal level of experience during different activities as well as a state of effortless concentration and enjoyment (29, 30). Flow theory suggests when individuals are in the state of flow, they want to maintain the state (29). Flow in gaming refers to player enjoyment in the process of playing videogames (31, 32). Flow experience is also regarded as the highest level of intrinsic motivation which contributes to the maintenance of online game behavior (33). Individuals playing online games while in the state of flow tend to continue or escalate gaming behaviors (34). Flow can also comprise a loss of self-consciousness and lead to time distortion (35). The model of game flow comprises eight elements including concentration, challenge, skills, control, clear goals, feedback, immersion, and social interaction which may be factors by which players distinguish high-rating games and low-rating games and help game developers to attract gamers (31, 35).

Some studies have investigated the relationship between flow experience and GD (36–38). Flow experience as an emotional state may embrace perceptional distortion and enjoyment, which has been found to have a stronger impact in maintaining gaming addiction than repetition of other favorite activities (39). In addition, time loss is reported as one of the best indications of flow experiences (40). Given that addictive behaviors are related to constant rewards (41), flow as an optimal experience may be a contributory cause of addictive behaviors when engaging in an activity (e.g., gaming disorder, social media addiction). Moreover, one study reported that online flow mediated the relationship between social games [i.e., Massively Multiplayer Online Role Playing Games (MMORPGs) and Multiplayer Online Battle Arena (MOBA) games] and IGD (37). Flow is also experienced more by males and individuals with internet addition than females and non-internet addicted individuals (42).

### Time Spent Gaming

In the fifth edition of the Diagnostic and Statistical Manual of Mental Disorders (DSM-5), nine criteria were proposed for IGD including the need to spend increasing amounts of time gaming (i.e., tolerance) and unsuccessful attempts to control gaming activities [i.e., reduce time spent gaming or stopping gaming; (43, 44)]. Individuals who play videogames to escape spend more game time playing MMORPGs (45). In addition, time spent gaming may mediate the relationship between MMORPG and GD (46). Players who spend a large amount of time spent gaming may feel anxiety due to time loss (47). Greater amounts of gaming and time distortion may be risk factors for IGD (48, 49).

Time spent gaming has also been found to be associated with negative consequences among adolescents playing MMORPGs (e.g., less sleep and poor academic performance), but was a weaker influence on negative consequences in comparison with gaming motivations (e.g., escape reality and gain status) (50, 51). In addition, higher time spent gaming has been found to increase the probability of depression, musculoskeletal problems, and psychosomatic symptoms (52). Immersion motivation (i.e., game flow) may mediate the relationship between time spent gaming and well-being (53). Time spent gaming and game motivation are considered as less clinically specific outcomes variables (26). Although most individuals with gaming disorder spend excessive amounts of time gaming, not all excessive gaming is problematic (54).

### Fear of Missing Out

Fear of missing out (FoMO) has been defined as “a pervasive apprehension that others might be having rewarding experiences from which one is absent” [(55), p. 1,841]. FoMO is relatively stable personality traits (55). FoMO has been posited as comprising two dimensions (trait-FoMO and state-FoMO) that is not only reflection of a specific personal predisposition, but also a specific cognition in which individuals fear missing out on something or an experience when online (56). Moreover, FoMO is associated with some personality traits (e.g., neuroticism and narcissism) (57, 58), which is similar to the relationship between GD and personality traits (59).

A recent study indicated that social identity may influence online gaming addiction through the mediation of FoMO among MMORPG gamers (60). In addition, FoMO has also been found to mediate the relationship between healthy anxiety and both GD and problematic smartphone use (PSU) during the COVID-19 pandemic (61). State-FoMO have been found to indirectly predict IGD through avoidance expectancies among a sample of German and Spanish respondents (56). Based on the self-determination theory, FoMO should be associated with increased GD severity to meet social needs (61). Moreover, based on the I-PACE model, FoMO as one an internet-related cognitive bias (state-FoMO) and a specific personality trait (trait-FoMO) may impact on GD (6).

### Network Analysis

Network analysis has been increasingly used in social and physical sciences over the past two decades (62). The basic assumptions and explanatory model of relevant variables may be provided through using network analysis in psychopathology. The advantage of network analysis lies in its graphic visualization, which may describe the relationship between causal variables more intuitively using graph theory. In addition, network analysis may also better explain the causal interaction of an episode of a mental health disorder and track the time change of nodes and edges as opposed to unclear latent variables analysis (63). Some research has explored behavioral addictions utilizing a network analysis perspective. For example, in an internet addiction study, defensive and secretive behaviors and concealment of internet use were identified as the core symptoms of internet addiction among Japanese youth with autism spectrum disorder (64). In addition, network analysis showed that withdrawal and preoccupation were the core symptoms of smartphone addiction using the short version of smartphone addiction scale (SAS-SV) among Brazilian adolescents by providing the visualization of network structure of smartphone addiction by facet-level network and item-level network analysis (i.e., strong and weak connections between symptoms) (65). Moreover, in a study among Chinese grade four and grade eight students, item-level network analysis showed that loss of control and continued excessive use were identified as the core symptoms of problematic smartphone use (PSU) utilizing the Smartphone Addiction Proneness Scale and also found the same global structure between the grade four group and grade eight group (66). Network analysis has also been used to visualize the structure of other psychiatric or related diseases (e.g., competitive state anxiety and post-traumatic stress disorder) (67, 68). However, few studies to date have explored the structure of GD using network analysis, especially the relationship between GD and related variables.

### The Present Study

Although the relationship between GD, game motivation, time spent gaming and/or FoMO has been studied utilizing correlation analysis or mediation/moderation effect analysis, the network analysis approach has rarely been utilized for better understanding the relationships between GD, self-compensation motivation, game flow, time spent gaming, and FoMO. Therefore, network analysis was used in the present study to better explain the relationship between the aforementioned variables among a sample of Chinese gamers. Moreover, a visual network analysis may contribute to exploring the inner relationship between GD, self-compensation motivation, game flow, time spent gaming and FoMO. In addition, gender differences among a sample of Chinese university students were also compared by the structure and global strength of the network.

## Methods

### Participants and Procedure

From October 2020 to December 2020, a total of 1,794 university students from two provinces in China (Jiangxi and Liaoning) participated in an online survey. The study adopted cross-sectional design and utilized convenience sampling through *Wenjuanxing* (a popular Chinese survey-hosting website). Among the initial participants, 159 were excluded from the analysis because they had not played videogames in the past year or did not complete all the survey items. The total sample of 1,635 participants comprised male university students (913, mean age = 19.76, *SD* = 1.59) and female university students (722, mean age = 19.55, *SD* = 1.45). The online survey took ~5 min to complete.

### Measures

#### Gaming Disorder

The Gaming Disorder Test (GDT) was used to assess the severity of gaming disorder (69). The GDT comprises four items assessing impaired control over gaming (i.e., “*I have had difficulties controlling my gaming activity”*), increasing priority to gaming (i.e., “*I have given increasing priority to gaming over other life interests and daily activities*”), continuation or escalation of gaming (i.e., “*I have continued gaming despite the occurrence of negative consequences”*), and marked distress or significant functional impairment [i.e., “*I have experienced significant problems in life (e.g., personal, family, social, education, occupational) due to the severity of my gaming behavior”*]. Items are responded to on a five-point scale ranging from “*never”* (1) to “*very often”* (*5*). Higher scores indicate a higher risk of gaming disorder. In the present study, the McDonald's ω and Cronbach's alpha were 0.85 and 0.84.

#### Self-Compensation Motivation

Self-compensation motivation was assessed using the Self-Compensation Motivation Questionnaire (SCMQ) (20), and has also been used to assess online game motivation (28). The SCMQ comprises three items: (i) “*When I encounter setbacks, I try to play videogames and then feel better”*; (ii) “*When I play videogames, I may get some material compensation (e.g., game currency and game rank)”*; (iii) “*When I play videogames, I may get some psychological compensation.”* Items are responded to on a five-point scale ranging from “*totally disagree”* (1) to “*totally agree”* (*5*). Higher scores indicate a higher self-compensation motivation toward online gaming. In the present study, the McDonald's ω and Cronbach's alpha were 0.87 and 0.87.

#### Game Flow

Game flow was assessed using the Game Flow Questionnaire (GFQ) modified from previous studies (33, 70). The GFQ includes five items: (i) “*I forgot about my immediate surroundings when I play online game*”; (ii) “*After playing online game, I felt like I came back to the ‘real world' after a journey*”; (iii) “*When I play online game, my body is in the room, but my mind is inside the world created by the game*”; (iv) “*I feel flexible when I play online games*”; (v) “*Playing online game was fun.”* Items are responded to on a five-point scale ranging from “*totally disagree”* (1) to “*totally agree”* (*5*). Higher scores indicate a higher level of game flow. The McDonald's ω and Cronbach's alpha were 0.79 and 0.78 in the present study.

#### Time Spent Gaming

Time spent gaming comprised four items relating to how many years they had been gaming (i.e., years spent gaming), how many days a week they spent gaming (i.e., gaming days per week), how many hours they spent gaming on weekdays (i.e., weekday game hours), and how many hours they spent gaming at the weekend (i.e., weekend game hours). All items were responded to on a three-point scale. For years spent gaming, 3 years or fewer = 1, 4–10 years = 2, more than 10 years = 3. For gaming days per week, 2 days or fewer = 1, 3–5 days = 2, 6 days or more = 3. For weekday and weekend game hours, 3 hours or fewer = 1, 4–10 h = 2, 10 h or more = 3. The McDonald's ω and Cronbach's alpha of four items were 0.73 and 0.68 in the present study.

#### Fear of Missing Out

The Trait-State Fear of Missing Out Scale (T-SFoMOS) was used to assess fear of missing out (56). The Chinese version of T-SFoMOS (T-SFoMOSC) has good reliability and validity (71). The T-SFoMOSC has 12 items and two dimensions, comprising trait-FoMO (e.g., “*I get worried when I find out my friends are having fun without me”*) and state-FoMO [e.g., “*When I have a good time it is important for me to share the details online (e.g., updating status)”*], which is in line with the Wegmann's et al. (56). T-SFoMOS. Items are responded to on a five-point scale ranging from “*totally disagree”* (1) to “*totally agree”* (*5*). A higher total score indicates a greater level of FoMO. In the present study, the McDonald's ω and Cronbach's alpha were 0.90 and 0.89 (total scale), 0.87 and 0.86 (trait-FoMO), and 0.85 and 0.85 (state-FoMO), respectively.

### Statistical Analysis

Descriptive statistics, effect size (Cohen's *d*), network analysis, Cronbach's alpha, McDonald's ω and correlation analysis (i.e., Pearson and Bayesian) were conducted utilizing JASP (Jeffrey's Amazing Statistics Program). R package was used to perform the network comparison test (NCT) on gender (72).

EBICglasso model with at least absolute shrinkage and selection operator (LASSO) (73) and based on the Extended Bayesian Information Criterion (EBIC) (74) was utilized to assess network characteristics and structure. The tuning parameter was set 0.5 for the EBICglasso to ensure a greater sensitive and specific network structure. Nodes represent study variables and edges represent correlation between two nodes, which comprise network system. The centrality index describes the relationship between multiple nodes including betweenness, closeness, and strength (75). The correlation stability coefficient (*CS*-coefficient) was calculated to indicate centrality stability of node, which is preferable to at least 0.25 and better with more than 0.5 (76). Blue lines represent positive partial correlations between nodes/variables, while red lines represent negative partial correlations. Edge thickness and darkness indicate the association strength between nodes/variables. Bootstrapping (1,000 times) with 95% confidence intervals was calculated to estimate edge stability. The network structure and global network strengths between gender were compared through the network comparison test (NCT) (77).

### Ethics

The study was examined and approved by the research team's University Research Ethics Committee, and complied with the Declaration of Helsinki. All participants provided informed consent.

## Results

### Descriptive Statistics and Correlation Analyses

In Table 1, descriptive statistics of the total sample and comparison of the study variables between gender were shown. There were significant differences between gender for gaming disorder (*t* = 10.072, *p* < 0.001, Cohen's *d* = 0.502), self-compensation motivation (*t* = 6.389, *p* < 0.001, Cohen's *d* = 0.318), years spent gaming (*t* = 13.586, *p* < 0.001, Cohen's *d* = 0.677), gaming days per week (*t* = 12.894, *p* < 0.001, *Cohen's d* = 0.642), weekday gaming hours (*t* = 10.391, *p* < 0.001, Cohen's *d* = 0.518), and weekend gaming hours (*t* = 13.896, *p* < 0.001, Cohen's *d* = 0.692). Self-compensation motivation, game flow, FoMO and time spent gaming were all significantly and positively associated with GD (all *p* < 0.01; Supplementary Appendix 1). The Bayesian correlation showed that all of log(BF_10_) were more than 3, which further verified the statistically significant correlations between GD and other variables.

**Table 1:** Descriptive characteristics of the sample (*n* = 1,635).

**Variables**	**Total** **(*n* = 1,635)**	**Males** **(*n* = 913)**	**Females** **(*n* = 722)**	***t***	***p***	**Cohen's *d***
Age (years)	19.67 ± 1.53	19.76 ± 1.59	19.55 ± 1.45	2.706	0.007	0.135
Gaming disorders	6.92 ± 2.72	7.51 ± 2.94	6.18 ± 2.21	10.072	<0.001	0.502
Self-compensation motivation	9.07 ± 2.69	9.44 ± 2.73	8.60 ± 2.56	6.389	<0.001	0.318
Game flow	14.66 ± 3.71	14.91 ± 3.86	14.36 ± 3.49	2.972	0.003	0.148
FoMO	27.36 ± 8.57	26.59 ± 8.47	28.33 ± 8.60	4.097	<0.001	0.167
Trait-FoMO	11.26 ± 4.24	10.95 ± 4.16	11.66 ± 4.32	3.345	<0.001	0.167
State-FoMO	16.09 ± 5.31	15.64 ± 5.29	16.67 ± 5.29	3.930	<0.001	0.196
Years spent gaming	5.90 ± 3.95	7.02 ± 3.87	4.48 ± 3.58	13.586	<0.001	0.677
Gaming days per week	3.46 ± 2.42	4.11 ± 2.38	2.63 ± 2.20	12.894	<0.001	0.642
Weekday gaming hours	2.20 ± 2.68	2.79 ± 3.20	1.45 ± 1.53	10.391	<0.001	0.518
Weekend gaming hours	3.39 ± 3.26	4.33 ± 3.53	2.20 ± 2.42	13.896	<0.001	0.692

### EBICglasso Network Analysis

**Figure 1:**
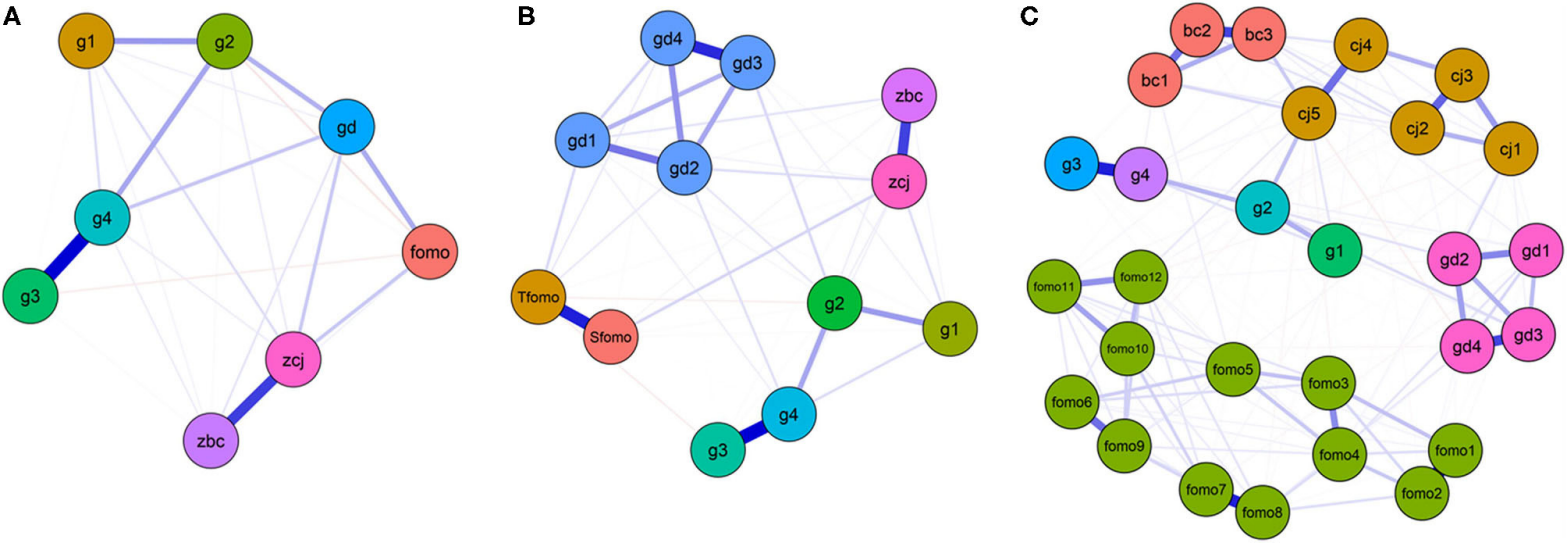
EBICglasso model based on network analysis according to the relationships between gaming disorder (GD), compensation motivation, game flow, time spent gaming, and fear of missing out (FoMO) among 1,635 participants in domain-level network **(A)**, in facet-level network **(B)**, and in item-level network **(C)**. gd, gd1~gd4, Gaming disorder; zbc, bc1~bc3, self-compensation motivation; zcj, cj1~cj5, game flow; fomo, fomo1~fomo12, FoMO; Tfomo, trait-FoMO; Sfomo, state-FoMO; g1~g4, game time.

The EBICglasso domain-level network including self-compensation motivation, game flow, time spent gaming, FoMO and GD are shown in Figure 1A. Nodes weekday gaming hours and weekend gaming hours had the strongest edge intensity (*r* = 0.632). Self-compensation motivation and game flow had a strong edge intensity (*r* = 0.476). Node GD had a direct association with FoMO intensity (*r* = 0.205), game flow (*r* = 0.129), gaming days per week (*r* = 0.188), and weekend gaming hours (*r* = 0.137) (Supplementary Appendix 2). The *CS*-coefficients of GD, self-compensation motivation, game flow, four of game times, and FoMO were 0.75, 0.66, 0.90, 0.50, 0.79, 0.71 1.10, and 0.49, respectively (Supplementary Appendix 3). Weekend gaming hours (strength = 1.885) and GD (betweenness = 1.755, closeness = 1.796) were the most central nodes (Supplementary Appendix 4).

Facet-level and item-level of the network including nodes self-compensation motivation, game flow, time spent gaming, FoMO and GD data are shown in Figures 1B,C. GD was connected with self-compensation motivation, game flow, time spent gaming and FoMO in the network system. In the facet-level network, nodes weekday gaming hours and weekend gaming hours had also the strongest edge intensity (*r* = 0.627), while trait-FoMO and state-FoMO (*r* = 0.551), gd3 (“*continuation or escalation of gaming”*) and gd4 (“*gaming problems”*) (*r* = 0.524), as well as self-compensation motivation and game flow (*r* = 0.464) also had strong edge intensity (Supplementary Appendix 5). Weekend gaming hours had the highest level of strength centrality (1.422). Nodes gd2 (“*increasing priority to gaming”*) and gd3 (“*continuation or escalation of gaming”*) had higher strength centrality (1.326 and 1.151) (Supplementary Appendix 6). In the item-level network, nodes gaming days per week and gd2 (“*increasing priority to gaming*”) had higher betweenness (1.702 and 1.256) and closeness (1.631 and 1.175) centrality, while node bc3 (“*playing online games can make me get psychological compensation”*) had the highest level of strength centrality (1.265), and fomo2 (“*I fear my friends have more rewarding experiences than me”*) had higher strength centrality (1.073) (Supplementary Appendix 7).

### EBICglasso Network Analysis for Males and Females

In the domain-level network, the edge of weekday gaming hours and weekend gaming hours had the strongest intensity among males (*r* = 0.680), while self-compensation motivation and game flow had the strongest intensity among females (*r* = 0.445). Weekend gaming hours had the highest strength coefficient among males (1.743) and game flow had the highest strength coefficient among females (1.403). The *CS*-coefficients of GD, self-compensation motivation, game flow, the four different times spent gaming, and FoMO among males and females were 0.73 and 0.58, 0.61 and 0.58, 0.96 and 0.83, 0.41 and 0.33, 0.63 and 0.72, 0.71 and 0.53, 1.10 and 0.72, and 0.42 and 0.37, respectively. In the facet-level network, the edge of weekday gaming hours and weekend gaming hours had the strongest edge intensity among males (*r* = 0.666), while trait-FoMO and state-FoMO had the strongest edge intensity among females (*r* = 0.533). Node gd2 (“*increasing priority to gaming”*) had the highest level of strength centrality among males (1.421), while node gd3 (“*continuation or escalation of gaming”*) had the highest level of strength centrality among females (1.509). In the item-level network, item gd2 (“*increasing priority to gaming*) had the highest level of strength centrality among males (1.377) and item fomo1 (“*I fear others have more rewarding experiences than me”*) had the highest level of strength centrality among females (1.371) (Supplementary Appendices 8–20).

### Comparison of Network Between Males and Females

The domain-level network structure had a significant difference between gender according to the network comparison test (NCT) (*M* = 0.309, *p* = 0.001). However, the global strengths had no significant difference between males and females (2.33 vs. 2.81, *p* = 0.067).

## Discussion

The present study has verified that GD (based on the GDT score) among males was significantly higher than females, which is in line with previous studies (78–80). In addition, self-compensation motivation and time spent gaming were also significantly higher among males. Some studies have proposed that need for escapism and interpersonal relationships may drive online gaming addiction (81). In a previous Taiwanese study, male high school students spent longer times gaming and had stronger motivation or desire to play videogames than those of females for entertainment and interpersonal relationships (82). Males prefer to conceal negative emotions (e.g., anxiety and depression) and a minority adopt coping behaviors to escape real life issues and to relieve negative emotion (i.e., self-compensation motivation) that can sometimes have negative consequences (e.g., gaming, drinking alcohol, and smoking cigarettes) (22). Moreover, among massively multiplayer online gamers, social motivation may predict addictive gaming, which is also regarded as a form of social compensation for relieving feelings of isolation and social anxiety (83). In the present study, GD was associated with game motivation and time spent gaming as well as FoMO, which is consistent with previous research [e.g., (26, 61)].

In the domain-level and facet-level network analysis for the present study, weekday gaming hours and weekend gaming hours had the strongest edge intensity. Based on the ICD-11, one of the main manifestations of GD is the impaired control over gaming, including onset, frequency, intensity, duration, termination, and context (1). In addition, the DSM-5 lists nine criteria for IGD including tolerance, which represents an increase in time spent playing for satisfying a growing desire and need for gaming (84). Although some scholars have questioned the characteristic of tolerance in IGD because increased time is not necessarily a good indicator of problematic gamimg (54, 85), individuals with GD clearly spend more time gaming than non-problematic gamers.

In the facet-level network, Nodes gd2 (“*increasing priority to gaming”*) and gd3 (“*continuation or escalation of gaming”*) had higher strength centrality alongside weekend gaming hours. The results imply that individuals with GD excessively engage in videogames to satisfy psychological needs, spend more weekend time gaming, ignore other life interests, give up daily activities, and experience negative outcomes (e.g., poor sleep, poor academic performance, and maladaptive interpersonal relationships). In the item-level network, node bc3 (“*Playing online games can make me get psychological compensation”*) and fomo2 (“*I fear my friends have more rewarding experiences than me”*) had the higher level of strength centrality than other nodes. These findings indicate the importance of game compensation motivation and trait-FoMO in the GD-related network. Based on compensation theory (86), individuals play videogames to satisfy their needs and to compensate themselves (through escapism and achievement motivation) when facing difficult situations in their life. Moreover, as a relatively stable personality characteristic, trait-FoMO is associated with GD like other personality traits (e.g., high neuroticism, low agreeableness, and low conscientiousness) (18). Elhai et al. (87) found that FoMO was an important variable that may explain GD based on trait theory. According to the self-determination theory (88), need deficits could contribute to poor mental health and show a general sensitivity to fear of missing out on something and/or rewarding experiences (55).

In addition, node centrality stability was robust in both the total network and gender network which may provide further support for the correlational relationship between GD, self-compensation motivation, game flow, time spent gaming, and FoMO. Gamers with high level of trait-FoMO may be driven by self-compensation motivation and game flow (the highest level of intrinsic motivation) and spend more time gaming, which may lead to GD for a minority of gamers. A vicious cycle may also occur in which GD may trigger more negative emotion including FoMO and depression (61, 89), reinforce game motivation, and increase time spent gaming.

The findings of the present study may help in the development of treatment of IGD. Some scholars have studied the treatment of IGD for possible or best interventions to decrease game immersion and time spent in gaming behavior. A systematic review using meta-analysis indicated that cognitive-behavioral therapy (CBT) had high efficacy in reducing IGD symptoms (90). Another systematic review including medication, CBT and other interventions and psychosocial treatments reported inconsistent treatment outcomes and emphasized the importance of well-designed and adequately powered clinical trial (91). Torres-Rodríguez et al. (92) designed an Individualized intervention program (Programa Individualizado Psicoterapéutico para la Adicción a las Tecnologías de la información y la comunicación, PIPATIC) for 12–18 years old adolescents with IGD, which included psychoeducational, regular treatment, intrapersonal, interpersonal, family intervention, and development of a new lifestyle. The PIPATIC treatment program was found to be more effective than CBT in successfully treating IGD (93).

In China (where the present study was based), the Expert Consensus on the Prevention and Treatment of GD in China (5) introduced the definition, clinical features, risky factors, diagnosis and evaluation for GD (5). In addition, in the ECPTGDC (5), personalized integrated intervention including psychological and pharmacological treatments and other treatment modalities was recommended and supervision and coordination of medical care, school, family, and society were also needed (94). In the future, effective prevention and intervention for GD should be developed in China and around the world.

There were several study limitations in the present study. First, the use of convenience sampling meant the study lacks representativeness. Second, the utilization of cross-sectional data means that causal relationships between the study variables cannot be determined. Third, self-report data collected via the survey may lead to various methods biases (e.g., social desirability and motivation preference). Fourth, the network analysis examined the relationship variables only at a specific time without time series study. Fifth, the present study did not provide a validated cut-off score to distinguish between individuals at risk of GD and those not at risk as the GDT has no established cut-off. In future studies, more representative sampling and longitudinal network analysis are recommended. In addition, due to the brevity of the survey (i.e., ~5 min to complete), sociodemographic characteristics such as income and type of course taken at the university, and possible stressors or social vulnerabilities and psychiatric comorbidity were not examined. Therefore, the aforementioned variables need to be investigated in future research.

## Conclusions

The relationships between gaming disorder (GD), self-compensation motivation, game flow, time spent gaming, and FoMO were examined using a network analysis approach. The results indicated that time spent gaming at weekends and the total GD score were the core nodes in the domain-level relationship network between GD, self-compensation motivation, game flow, time spent gaming, and FoMO. The network structure demonstrated significant gender differences among a sample of Chinese gamers. The results suggest that GD is closely associated with self-compensation motivation, game flow, time spent gaming, and FoMO. FoMO and gaming motivation (i.e., self-compensation and game flow) may increase time spent gaming and facilitate GD. Therefore, interventions that decrease game immersion and time spent gaming are likely to decrease GD.

## Data Availability Statement

The original contributions presented in the study are included in the article/Supplementary Material, further inquiries can be directed to the corresponding author/s.

## Ethics Statement

The studies involving human participants were reviewed and approved by Gannan Medical University Research Ethics Committee. The patients/participants provided their written informed consent to participate in this study.

## Author Contributions

LL and ZN: conceived and designed the study, collected the data, and wrote the first draft of the paper. LL and MG: analyzed and interpreted the data. SM: contributed reagents, materials, and analysis tool. MG: edited and contributed to the revised paper. All authors contributed to the article and approved the submitted version.

## Funding

This study was supported by Jiangxi University Humanities and Social Science Research Project XL20104, The Science Education Program Project Thirteenth Five-Year Plan of Jiangxi Province 2020GX184, Doctor start-up fund of Gannan Medical University QD201819, Key project of Gannan Medical University ZD201838, The International Innovation Team of Jilin University 2019GJTD06, and Humanities and Social Sciences Youth Fund of the Ministry of Education of China 21YJC630028.

## Conflict of Interest

The authors declare that the research was conducted in the absence of any commercial or financial relationships that could be construed as a potential conflict of interest.

## Publisher's Note

All claims expressed in this article are solely those of the authors and do not necessarily represent those of their affiliated organizations, or those of the publisher, the editors and the reviewers. Any product that may be evaluated in this article, or claim that may be made by its manufacturer, is not guaranteed or endorsed by the publisher.
