# Supplementary material for: Relationship Between Gaming Disorder, Self-Compensation Motivation, Game Flow, Time Spent Gaming, and Fear of Missing Out Among a Sample of Chinese University Students: A Network Analysis
Source: Front Psychiatry. 2021 Nov 1;12:761519. doi: 10.3389/fpsyt.2021.761519 (PMC8591052; doi:10.3389/fpsyt.2021.761519)
Supplement: Supplementary file 1 [file Data_Sheet_1.docx]

**Supplementary materials**

**Appendix S1.** Correlation analysis of the study variables

|  | Gaming disorder | Self-compensation motivation | Game flow | FoMO | Trait-FoMO | State-FoMO | Game time (years) | Weekly time spent gaming (days) | Everyday time spent gaming (hours) |
| --- | --- | --- | --- | --- | --- | --- | --- | --- | --- |
| Self-compensation motivation | 0.253** | — |  |  |  |  |  |  |  |
| log(BF_10_) | 50.655 | — |  |  |  |  |  |  |  |
| Game flow | 0.313** | 0.549** | — |  |  |  |  |  |  |
| log(BF_10_) | 80.611 | 288.857 | — |  |  |  |  |  |  |
| FoMO | 0.255** | 0.165** | 0.229** | — |  |  |  |  |  |
| log(BF_10_) | 51.513 | 19.024 | 40.529 | — |  |  |  |  |  |
| Trait-FoMO | 0.277** | 0.133** | 0.187** | 0.869** | — |  |  |  |  |
| log(BF_10_) | 61.555 | 10.985 | 25.587 | ∞ | — |  |  |  |  |
| State-FoMO | 0.191** | 0.160** | 0.220** | 0.919** | 0.603** | — |  |  |  |
| log(BF_10_) | 26.738 | 17.719 | 37.059 | ∞ | 365.073 | — |  |  |  |
| Game time (years) | 0.207** | 0.168** | 0.209** | 0.014 | 0.025 | 0.003 | — |  |  |
| log(BF_10_) | 32.128 | 19.825 | 32.888 | -3.310 | -2.964 | -3.467 | — |  |  |
| Weekly time spent gaming (days) | 0.336** | 0.183** | 0.221** | 0.006 | 0.008 | 0.004 | 0.368** | — |  |
| log(BF_10_) | 94.268 | 24.377 | 37.463 | -3.443 | -3.427 | -3.461 | 115.412 | — |  |
| Everyday time spent gaming (hours) | 0.222** | 0.159** | 0.135** | -0.020 | -0.034 | -0.005 | 0.203** | 0.304** | — |
| log(BF_10_) | 37.577 | 17.424 | 11.540 | -3.153 | -2.543 | -3.453 | 30.766 | 75.341 | — |
| gaming time spent on weekend (hours) | 0.331** | 0.215** | 0.232** | 0.032 | 0.013 | 0.040 | 0.280** | 0.425** | 0.697** |
| log(BF_10_) | 90.902 | 35.121 | 41.668 | -2.659 | -3.332 | -2.138 | 62.897 | 159.162 | ∞ |

Note: ** *p<*.01.

**Appendix S2.** Edge weight matrix of the domain-level network among total sample

|  | Gaming disorder | Self-compensation motivation | Game flow | FoMO | Game time 1 | Game time 2 | Game time 3 | Game time 4 |
| --- | --- | --- | --- | --- | --- | --- | --- | --- |
| Gaming disorder | 0.000 | 0.060 | 0.129 | 0.205 | 0.036 | 0.188 | 0.00 | 0.137 |
| Self-compensation motivation | 0.060 | 0.000 | 0.476 | 0.032 | 0.027 | 0.014 | 0.014 | 0.042 |
| Game flow | 0.129 | 0.476 | 0.000 | 0.131 | 0.078 | 0.047 | 0.000 | 0.039 |
| FoMO | 0.205 | 0.032 | 0.131 | 0.000 | -0.014 | -0.064 | -0.045 | 0.000 |
| Game time 1 | 0.036 | 0.027 | 0.078 | -0.014 | 0.000 | 0.252 | 0.007 | 0.083 |
| Game time 2 | 0.188 | 0.014 | 0.047 | -0.064 | 0.252 | 0.000 | 0.009 | 0.216 |
| Game time 3 | 0.00 | 0.014 | 0.000 | -0.045 | 0.007 | 0.009 | 0.000 | 0.632 |
| Game time 4 | 0.137 | 0.042 | 0.039 | 0.000 | 0.083 | 0.216 | 0.632 | 0.000 |


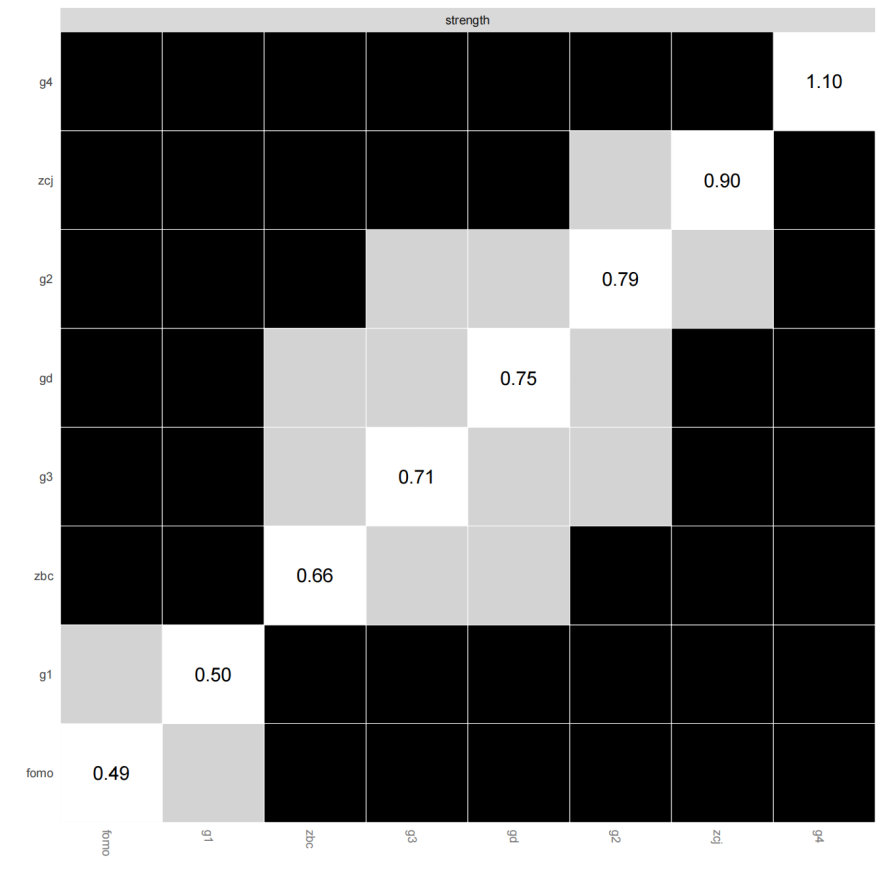


**Appendix S3.** Centrality stability (parametric) of the domain-level network among 1635 participants

**Appendix S4.** Centrality measures per variable of the domain-level network among 1635 participants

|  | Betweenness | Closeness | Strength |
| --- | --- | --- | --- |
| Gaming disorder | 1.755 | 1.796 | 0.056 |
| Self-compensation motivation | -0.845 | -1.313 | -0.364 |
| Game flow | 0.715 | -0.503 | 0.726 |
| FoMO | -0.845 | -0.303 | -1.186 |
| Game time 1 | -0.845 | -0.408 | -1.143 |
| Game time 2 | 0.195 | 1.080 | 0.200 |
| Game time 3 | -0.845 | -0.571 | -0.174 |
| Game time 4 | 0.715 | 0.223 | 1.885 |

**Appendix S5**. Edge weight matrix of the facet-level network among 1635 participants

| Variable | Sfomo | Tfomo | G1 | G2 | G3 | G4 | Gd1 | Gd2 | Gd3 | Gd4 | Zbc | Zcj |
| --- | --- | --- | --- | --- | --- | --- | --- | --- | --- | --- | --- | --- |
| Sfomo | 0.000 | **0.551** | -0.016 | -0.015 | 0.000 | 0.000 | 0.000 | 0.000 | 0.000 | 0.013 | 0.023 | 0.097 |
| Tfomo | 0.551 | 0.000 | 0.000 | -0.039 | -0.042 | -0.004 | 0.091 | 0.048 | 0.000 | 0.046 | 0.000 | 0.010 |
| G1 | -0.016 | 0.000 | 0.000 | 0.243 | 0.006 | 0.082 | 0.010 | 0.000 | 0.030 | 0.000 | 0.024 | 0.075 |
| G2 | -0.015 | -0.039 | 0.243 | 0.000 | 0.009 | 0.214 | 0.023 | 0.056 | 0.090 | 0.000 | 0.008 | 0.038 |
| G3 | 0.000 | -0.042 | 0.006 | 0.009 | 0.000 | **0.627** | 0.000 | 0.003 | 0.000 | 0.002 | 0.011 | 0.000 |
| G4 | 0.000 | -0.004 | 0.082 | 0.214 | 0.627 | 0.000 | 0.036 | 0.072 | 0.000 | 0.000 | 0.039 | 0.035 |
| Gd1 | 0.000 | 0.091 | 0.010 | 0.023 | 0.000 | 0.036 | 0.000 | **0.338** | 0.182 | 0.102 | 0.063 | 0.029 |
| Gd2 | 0.000 | 0.048 | 0.000 | 0.056 | 0.003 | 0.072 | 0.338 | 0.000 | 0.214 | 0.265 | 0.017 | 0.078 |
| Gd3 | 0.000 | 0.000 | 0.030 | 0.090 | 0.000 | 0.000 | 0.182 | 0.214 | 0.000 | **0.524** | 0.000 | 0.017 |
| Gd4 | 0.013 | 0.046 | 0.000 | 0.000 | 0.002 | 0.000 | 0.102 | 0.265 | 0.524 | 0.000 | 0.000 | 0.000 |
| Zbc | 0.023 | 0.000 | 0.024 | 0.008 | 0.011 | 0.039 | 0.063 | 0.017 | 0.000 | 0.000 | 0.000 | **0.464** |
| Zcj | 0.097 | 0.010 | 0.075 | 0.038 | 0.000 | 0.035 | 0.029 | 0.078 | 0.017 | 0.000 | 0.464 | 0.000 |

**Appendix S6.** Centrality measures per variable of the facet-level network among 1635 participants

| Variable | Betweenness | Closeness | Strength |
| --- | --- | --- | --- |
| Sfomo | -0.743 | -1.227 | -0.638 |
| Tfomo | 0.172 | -0.967 | -0.031 |
| g1 | -0.057 | -0.410 | -1.832 |
| g2 | 1.773 | 0.158 | -0.525 |
| g3 | -0.972 | -0.971 | -0.716 |
| g4 | 0.400 | -0.597 | **1.422** |
| gd1 | -0.057 | 1.191 | 0.186 |
| gd2 | 0.858 | 1.819 | **1.326** |
| gd3 | 0.400 | 1.187 | **1.151** |
| gd4 | -1.430 | 0.547 | 0.596 |
| zbc | -1.430 | -0.791 | -0.977 |
| zcj | 1.086 | 0.061 | 0.037 |

**Appendix S7**. Centrality measures per variable of the item-level network among 1635 participants

| Variable | Betweenness | Closeness | Strength |
| --- | --- | --- | --- |
| Bc1 | -0.608 | -0.889 | -0.646 |
| Bc2 | -0.811 | -0.791 | 0.415 |
| Bc3 | -0.446 | -0.385 | **1.265** |
| Cj1 | -0.081 | -0.031 | -0.815 |
| Cj2 | -1.013 | -0.486 | 0.050 |
| Cj3 | -0.041 | 0.175 | 0.339 |
| Cj4 | 0.203 | 0.941 | -0.398 |
| Cj5 | 3.283 | 2.054 | 0.894 |
| Fomo1 | -0.730 | -1.860 | 0.700 |
| Fomo10 | -0.567 | -0.094 | -0.542 |
| Fomo11 | -0.689 | 0.080 | -0.179 |
| Fomo12 | 1.297 | 0.528 | -0.076 |
| Fomo2 | -0.527 | -1.794 | **1.073** |
| Fomo3 | 0.041 | 0.060 | -0.039 |
| Fomo4 | 1.094 | 0.466 | 0.905 |
| Fomo5 | -0.446 | -0.587 | -1.414 |
| Fomo6 | -0.811 | -0.914 | -0.701 |
| Fomo7 | -0.486 | -0.611 | 0.675 |
| Fomo8 | 0.041 | -0.351 | 0.763 |
| Fomo9 | -0.405 | -0.524 | 0.489 |
| G1 | -1.135 | -0.830 | -3.130 |
| G2 | 1.702 | 1.631 | -0.758 |
| G3 | -1.135 | -0.869 | -1.729 |
| G4 | -0.081 | -0.116 | 0.954 |
| Gd1 | 1.054 | 1.175 | -0.517 |
| Gd2 | 1.256 | 1.335 | 0.911 |
| Gd3 | 0.081 | 1.474 | 0.792 |
| Gd4 | -0.041 | 1.212 | 0.719 |

**Appendix S8**. Edge weight matrix of the domain-level network between males and females

| Variable | Males | | | | | | | | Females | | | | | | | |
| --- | --- | --- | --- | --- | --- | --- | --- | --- | --- | --- | --- | --- | --- | --- | --- | --- |
|  | Fomo | G1 | G2 | G3 | G4 | Gd | Zbc | Zcj | Fomo | G1 | G2 | G3 | G4 | Gd | Zbc | Zcj |
| Fomo | 0.000 | 0.000 | 0.000 | -0.034 | 0.000 | 0.235 | 0.000 | 0.149 | 0.000 | 0.000 | -0.024 | 0.000 | 0.000 | 0.181 | 0.102 | 0.060 |
| G1 | 0.000 | 0.000 | 0.233 | 0.000 | 0.046 | 0.022 | 0.000 | 0.105 | 0.000 | 0.000 | 0.180 | 0.027 | 0.046 | 0.000 | 0.000 | 0.076 |
| G2 | 0.000 | **0.233** | 0.000 | 0.000 | 0.198 | 0.147 | 0.034 | 0.022 | -0.024 | **0.180** | 0.000 | 0.089 | 0.190 | 0.168 | 0.000 | 0.071 |
| G3 | -0.034 | 0.000 | 0.000 | 0.000 | 0.680 | 0.000 | 0.000 | 0.000 | 0.000 | 0.027 | 0.089 | 0.000 | 0.371 | 0.046 | 0.000 | 0.000 |
| G4 | 0.000 | 0.046 | 0.198 | **0.680** | 0.000 | 0.126 | 0.027 | 0.062 | 0.000 | 0.046 | 0.190 | **0.371** | 0.000 | 0.046 | 0.029 | 0.039 |
| Gd | **0.235** | 0.022 | 0.147 | 0.000 | 0.126 | 0.000 | 0.063 | 0.135 | **0.181** | 0.000 | 0.168 | 0.046 | 0.046 | 0.000 | 0.005 | 0.135 |
| Zbc | 0.000 | 0.000 | 0.034 | 0.000 | 0.027 | 0.063 | 0.000 | 0.490 | 0.102 | 0.000 | 0.000 | 0.000 | 0.029 | 0.005 | 0.000 | 0.445 |
| Zcj | 0.149 | 0.105 | 0.022 | 0.000 | 0.062 | 0.135 | **0.490** | 0.000 | 0.060 | 0.076 | 0.071 | 0.000 | 0.039 | 0.135 | **0.445** | 0.000 |

**Appendix S9**. Centrality measures per variable of the domain-level network between males and females

| Variable | Males | | | Females | | |
| --- | --- | --- | --- | --- | --- | --- |
|  | Betweenness | Closeness | Strength | Betweenness | Closeness | Strength |
| Fomo | -1.017 | -0.213 | -1.133 | -0.885 | -0.506 | -1.238 |
| G1 | -0.391 | -0.213 | -1.177 | -0.885 | -0.168 | -1.461 |
| G2 | 0.235 | 0.871 | -0.273 | 1.613 | 1.469 | 0.800 |
| G3 | -1.017 | -0.793 | 0.048 | -0.885 | -1.031 | -0.288 |
| G4 | 0.860 | 0.043 | **1.743** | 0.364 | -0.205 | 0.803 |
| Gd | 1.486 | 1.922 | 0.107 | 1.197 | 1.625 | -0.010 |
| Zbc | -1.017 | -1.318 | -0.354 | -0.677 | -0.826 | -0.009 |
| Zcj | 0.860 | -0.297 | 1.039 | 0.156 | -0.359 | **1.403** |


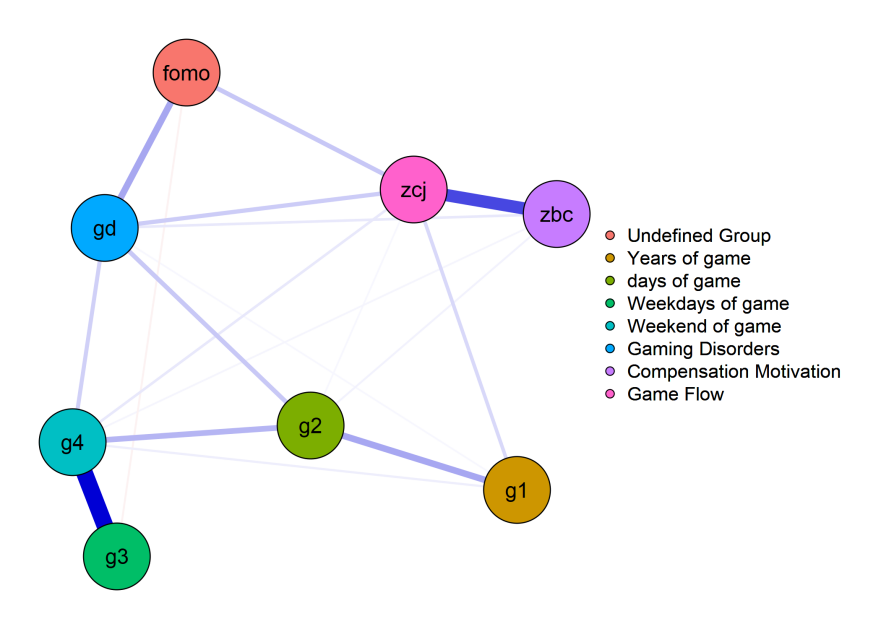

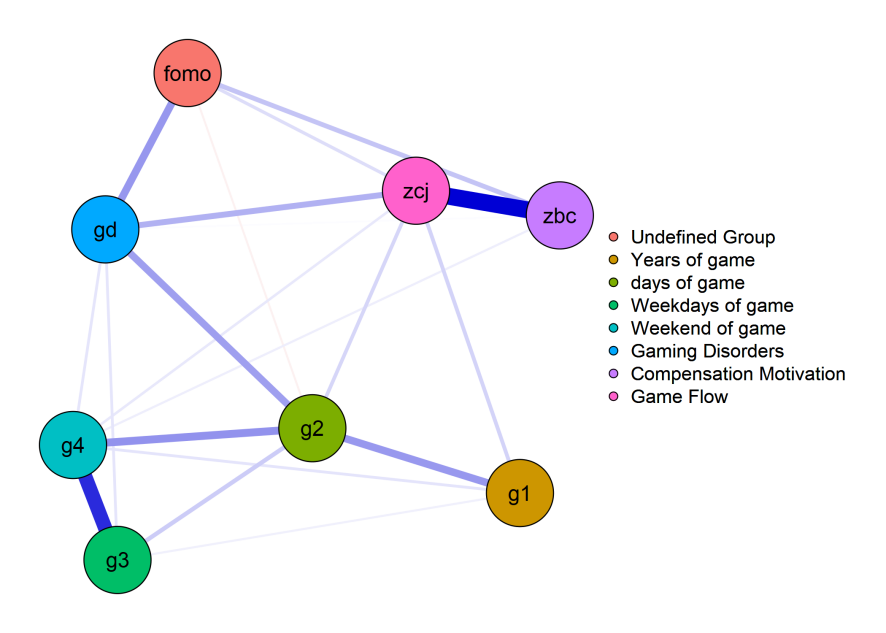


B

A

**Appendix S10.** Domain-level EBICglasso model based on network analysis according to the relationships between GD, self-compensation motivation, game flow, FoMO, and game time among 913 males(A) and 722 females (B). Note: gd = Gaming disorder, zbc = self-compensation motivation, zcj = game flow, fomo = Fear of missing out (FoMO), g1～g4= game time.


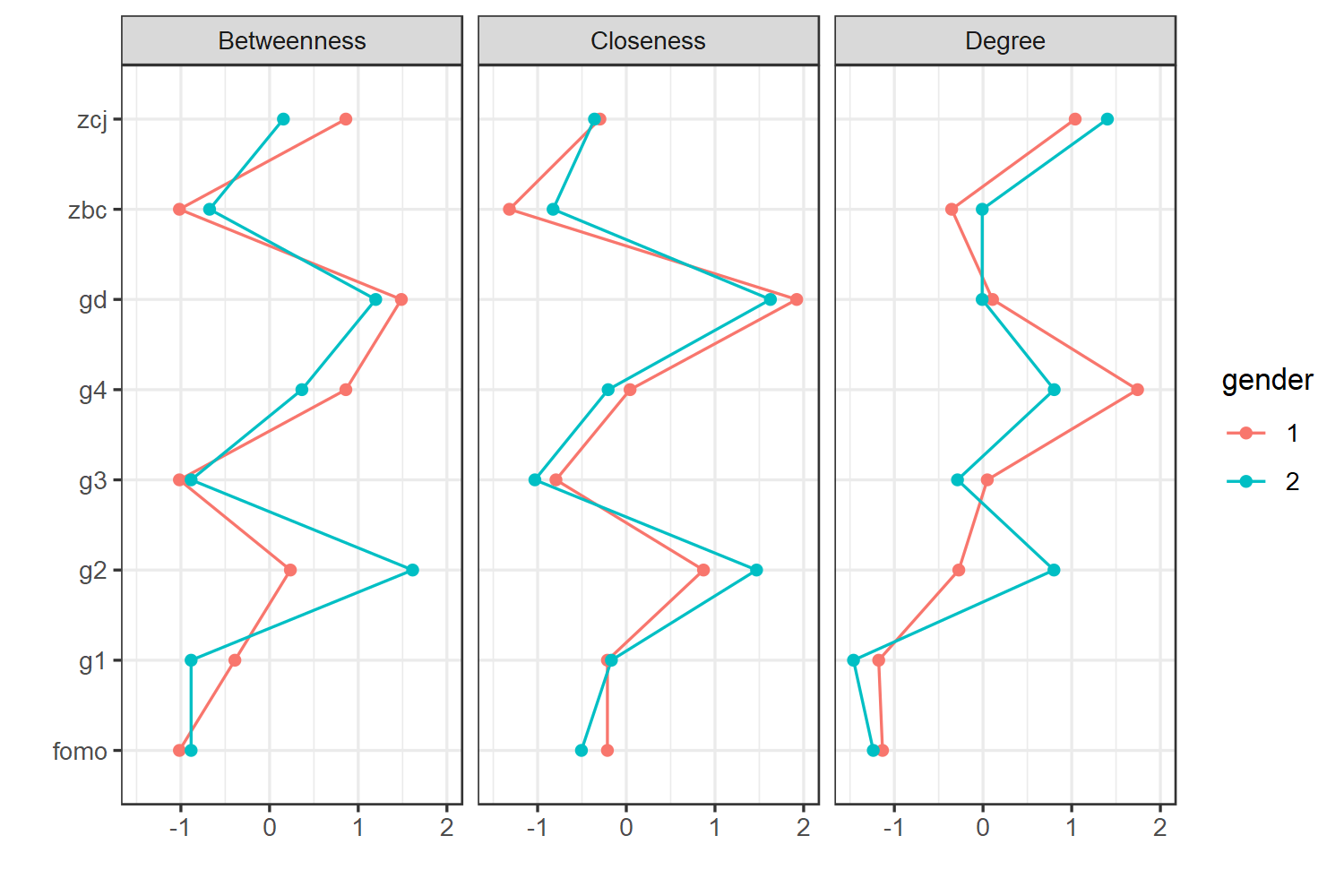


**Appendix S11.** Standardized estimates of node centrality in the domain-level network between males and females group. Note: 1 = male, 2 = female.


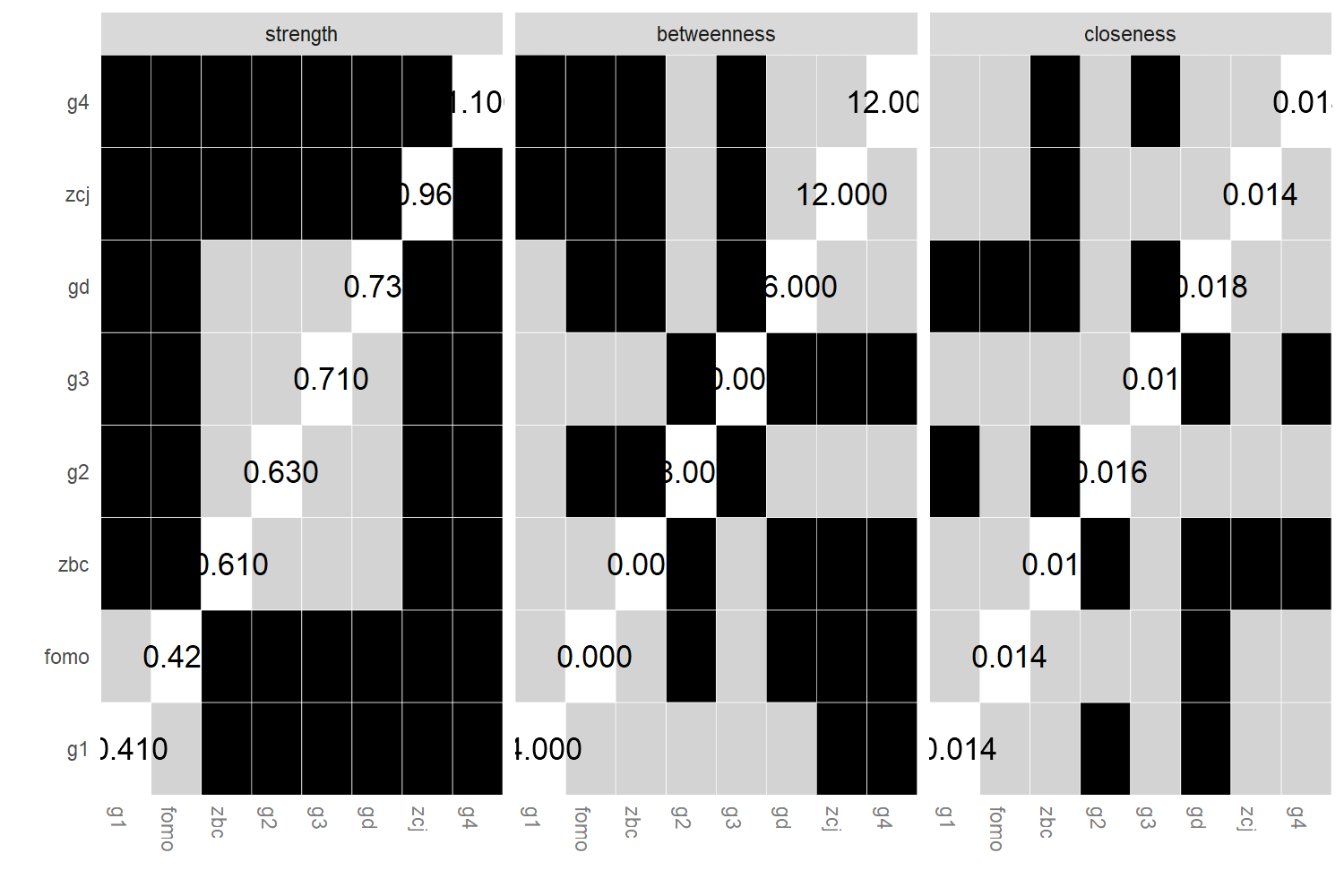

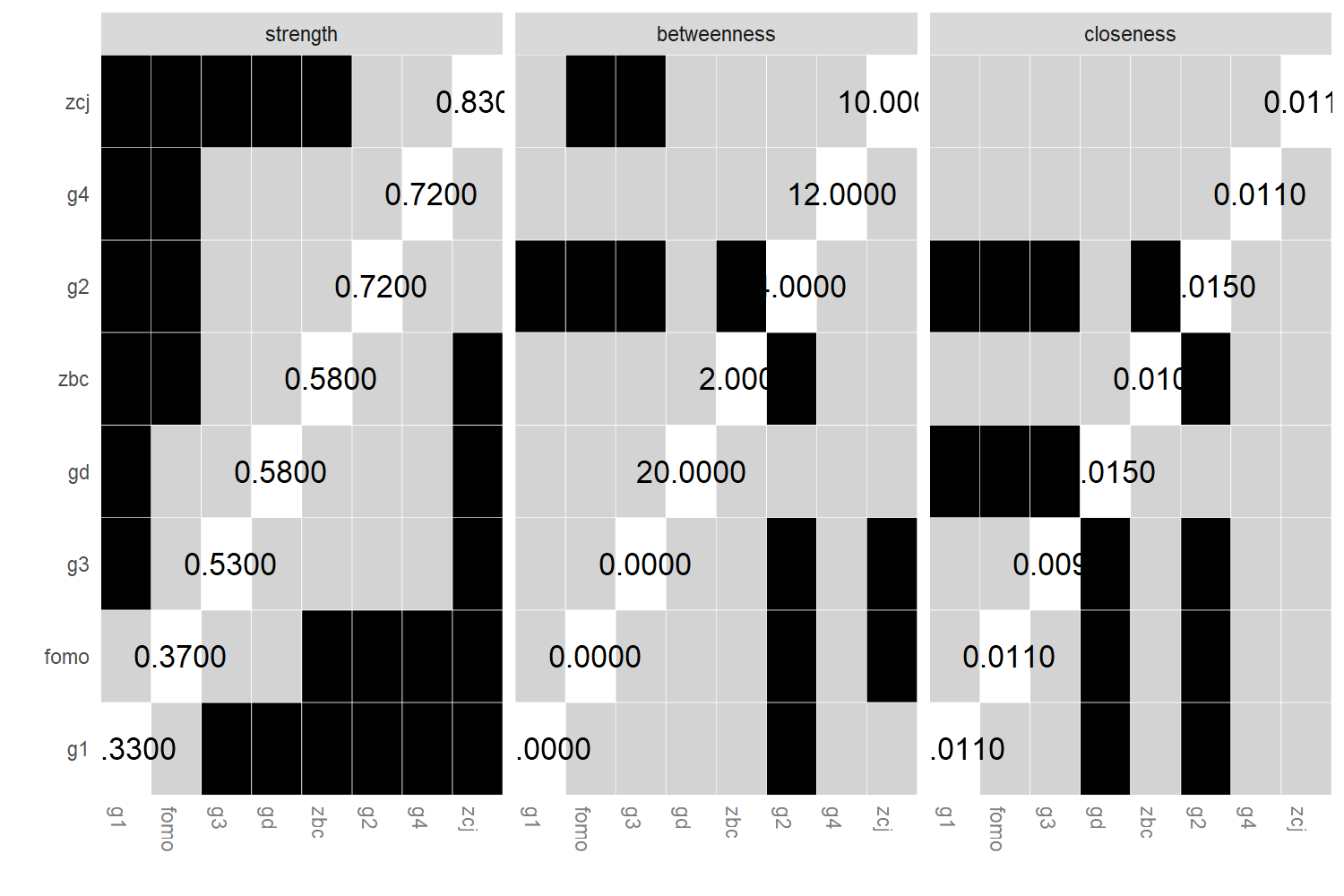


B

A

**Appendix S12.** Centrality stability (parametric) of the domain-level network between 913 males (A) and 722 females (B).

**Appendix S13**. Edge weight matrix of the facet-level network between males and females

| Variable | Males | | | | | | | | | | | | Females | | | | | | | | | | | |
| --- | --- | --- | --- | --- | --- | --- | --- | --- | --- | --- | --- | --- | --- | --- | --- | --- | --- | --- | --- | --- | --- | --- | --- | --- |
|  | Sfomo | Tfomo | G1 | G2 | G3 | G4 | Gd1 | Gd2 | Gd3 | Gd4 | Zbc | Zcj | Sfomo | Tfomo | G1 | G2 | G3 | G4 | Gd1 | Gd2 | Gd3 | Gd4 | Zbc | Zcj |
| Sfomo | 0.000 | 0.525 | 0.000 | 0.000 | 0.000 | 0.000 | 0.000 | 0.005 | 0.000 | 0.018 | 0.000 | 0.110 | 0.000 | 0.533 | 0.000 | -0.014 | 0.000 | 0.007 | 0.000 | 0.000 | 0.000 | 0.036 | 0.080 | 0.050 |
| Tfomo | **0.525** | 0.000 | 0.000 | 0.000 | -0.027 | 0.000 | 0.096 | 0.039 | 0.000 | 0.063 | 0.000 | 0.012 | **0.533** | 0.000 | 0.000 | -0.023 | -0.035 | 0.000 | 0.082 | 0.039 | 0.000 | 0.051 | 0.015 | 0.000 |
| G1 | 0.000 | 0.000 | 0.000 | 0.224 | 0.000 | 0.043 | 0.024 | 0.000 | 0.000 | 0.000 | 0.000 | 0.098 | 0.000 | 0.000 | 0.000 | 0.178 | 0.029 | 0.048 | 0.000 | 0.000 | 0.028 | -0.021 | 0.000 | 0.077 |
| G2 | 0.000 | 0.000 | 0.224 | 0.000 | 0.000 | 0.187 | 0.000 | 0.097 | 0.042 | 0.000 | 0.027 | 0.014 | -0.014 | -0.023 | 0.178 | 0.000 | 0.086 | 0.193 | 0.032 | 0.000 | 0.127 | 0.000 | 0.000 | 0.067 |
| G3 | 0.000 | -0.027 | 0.000 | 0.000 | 0.000 | 0.666 | 0.000 | 0.000 | 0.000 | 0.000 | 0.000 | 0.000 | 0.000 | -0.035 | 0.029 | 0.086 | 0.000 | 0.375 | 0.040 | 0.025 | 0.000 | 0.000 | 0.000 | 0.000 |
| G4 | 0.000 | 0.000 | 0.043 | 0.187 | **0.666** | 0.000 | 0.020 | 0.091 | 0.000 | 0.000 | 0.022 | 0.056 | 0.007 | 0.000 | 0.048 | 0.193 | **0.375** | 0.000 | 0.037 | 0.000 | 0.000 | 0.000 | 0.028 | 0.038 |
| Gd1 | 0.000 | 0.096 | 0.024 | 0.000 | 0.000 | 0.020 | 0.000 | 0.377 | 0.173 | 0.118 | 0.077 | 0.011 | 0.000 | 0.082 | 0.000 | 0.032 | 0.040 | 0.037 | 0.000 | 0.282 | 0.213 | 0.050 | 0.042 | 0.064 |
| Gd2 | 0.005 | 0.039 | 0.000 | 0.097 | 0.000 | 0.091 | 0.377 | 0.000 | 0.207 | 0.233 | 0.000 | 0.065 | 0.000 | 0.039 | 0.000 | 0.000 | 0.025 | 0.000 | 0.282 | 0.000 | 0.233 | 0.301 | 0.079 | 0.088 |
| Gd3 | 0.000 | 0.000 | 0.000 | 0.042 | 0.000 | 0.000 | 0.173 | 0.207 | 0.000 | 0.511 | 0.000 | 0.033 | 0.000 | 0.000 | 0.028 | 0.127 | 0.000 | 0.000 | 0.213 | 0.233 | 0.000 | 0.500 | 0.000 | 0.000 |
| Gd4 | 0.018 | 0.063 | 0.000 | 0.000 | 0.000 | 0.000 | 0.118 | 0.233 | **0.511** | 0.000 | 0.000 | 0.016 | 0.036 | 0.051 | -0.021 | 0.000 | 0.000 | 0.000 | 0.050 | 0.301 | **0.500** | 0.000 | -0.087 | 0.000 |
| Zbc | 0.000 | 0.000 | 0.000 | 0.027 | 0.000 | 0.022 | 0.077 | 0.000 | 0.000 | 0.000 | 0.000 | 0.475 | 0.080 | 0.015 | 0.000 | 0.000 | 0.000 | 0.028 | 0.042 | 0.079 | 0.000 | -0.087 | 0.000 | 0.433 |
| Zcj | 0.110 | 0.012 | 0.098 | 0.014 | 0.000 | 0.056 | 0.011 | 0.065 | 0.033 | 0.016 | **0.475** | 0.000 | 0.050 | 0.000 | 0.077 | 0.067 | 0.000 | 0.038 | 0.064 | 0.088 | 0.000 | 0.000 | **0.433** | 0.000 |

**Appendix S14**. Centrality measures per variable of the facet-level network between males and females

| **Variable** | **Males** | | | **Female** | | |
| --- | --- | --- | --- | --- | --- | --- |
|  | **Betweenness** | **Closeness** | **Strength** | **Betweenness** | **Closeness** | **Strength** |
| Sfomo | -0.463 | -1.159 | -0.652 | -0.572 | -1.382 | -0.369 |
| Tfomo | 0.013 | -0.939 | -0.171 | 0.025 | -1.240 | -0.085 |
| G1 | -0.463 | -0.283 | -1.861 | -1.020 | -0.568 | -2.034 |
| G2 | 0.013 | 0.261 | -0.949 | 1.965 | 0.662 | -0.362 |
| G3 | -0.939 | -0.915 | -0.487 | -1.020 | -1.130 | -1.008 |
| G4 | 0.648 | -0.299 | 1.298 | 0.174 | -0.686 | -0.335 |
| Gd1 | 0.965 | 1.685 | 0.433 | 0.473 | 0.757 | 0.236 |
| Gd2 | 2.393 | 2.147 | **1.421** | -0.572 | 1.044 | 1.243 |
| Gd3 | -0.939 | -0.010 | 0.744 | 1.816 | 1.551 | **1.509** |
| Gd4 | -0.939 | 0.075 | 0.719 | -0.871 | 0.986 | 1.245 |
| Zbc | -0.780 | -0.485 | -0.902 | -0.274 | -0.031 | -0.152 |
| Zcj | 0.489 | -0.078 | 0.408 | -0.124 | 0.038 | 0.112 |


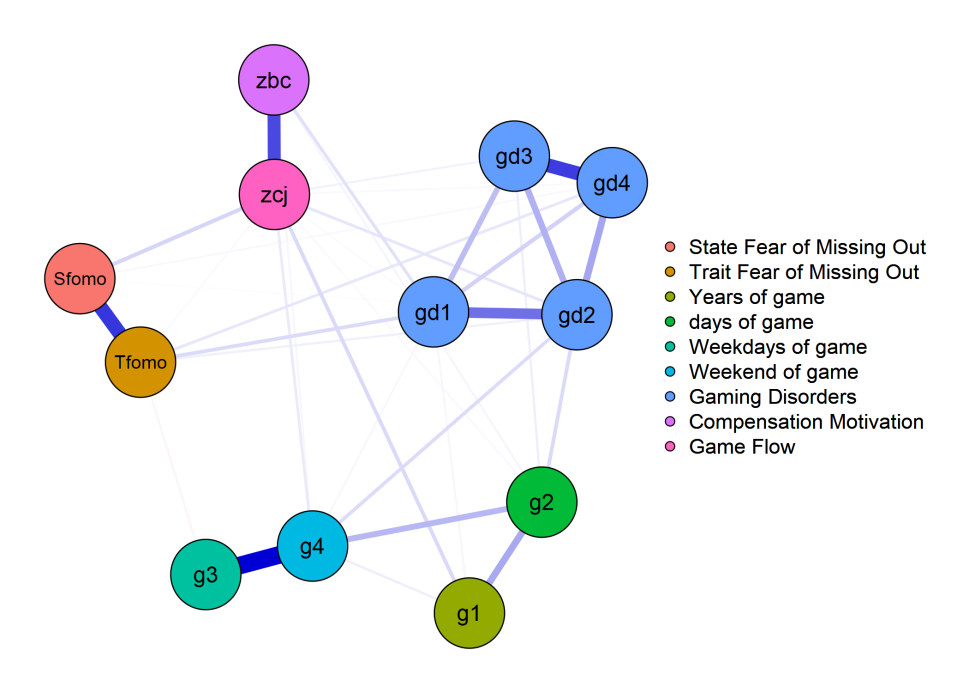

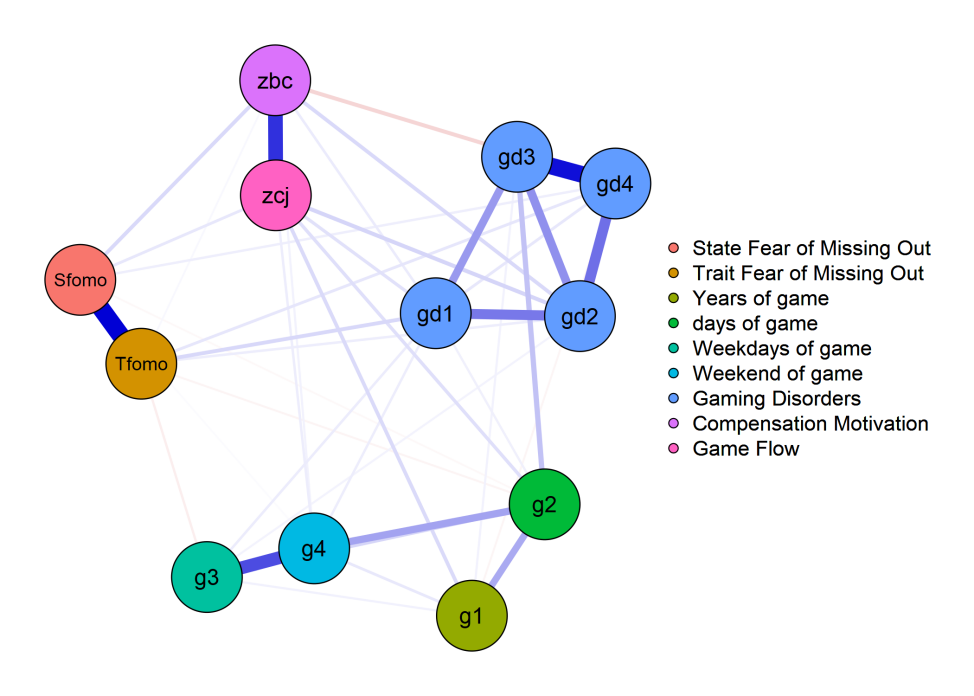


A

B

**Appendix S15.** Facet-level EBICglasso model based on network analysis according to the relationships between GD, self-compensation motivation, game flow, FoMO, and game time among 913 males(A) and 722 females (B). Note: gd1～gd4 = Gaming disorder, zbc = self-compensation motivation, zcj = game flow, Tfomo = trait-FoMO, Sfomo = state-FoMO, g1～g4= game time.


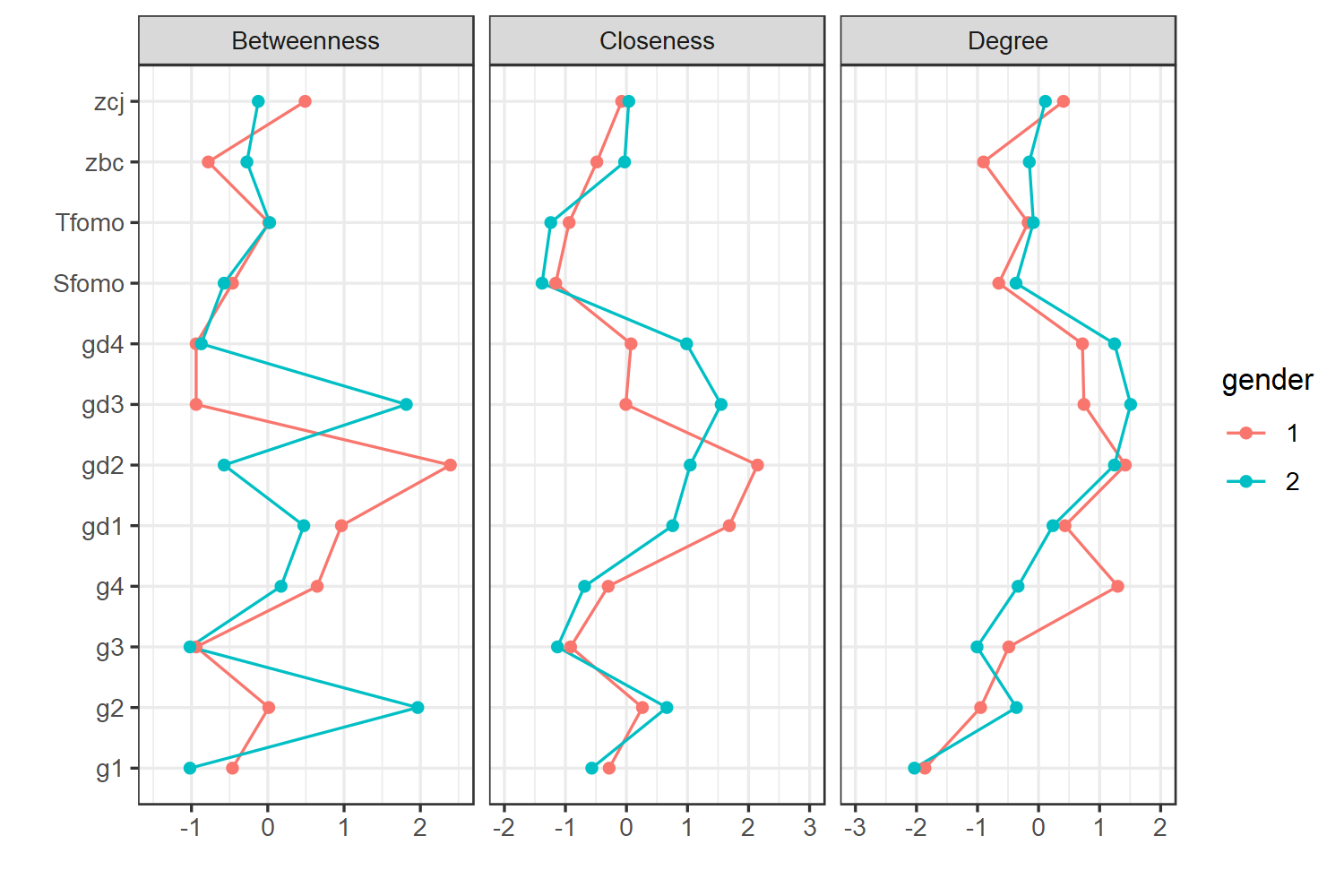


**Appendix S16.** Standardized estimates of node centrality in the facet-level network between males and females group. Note: 1 = male, 2 = female.


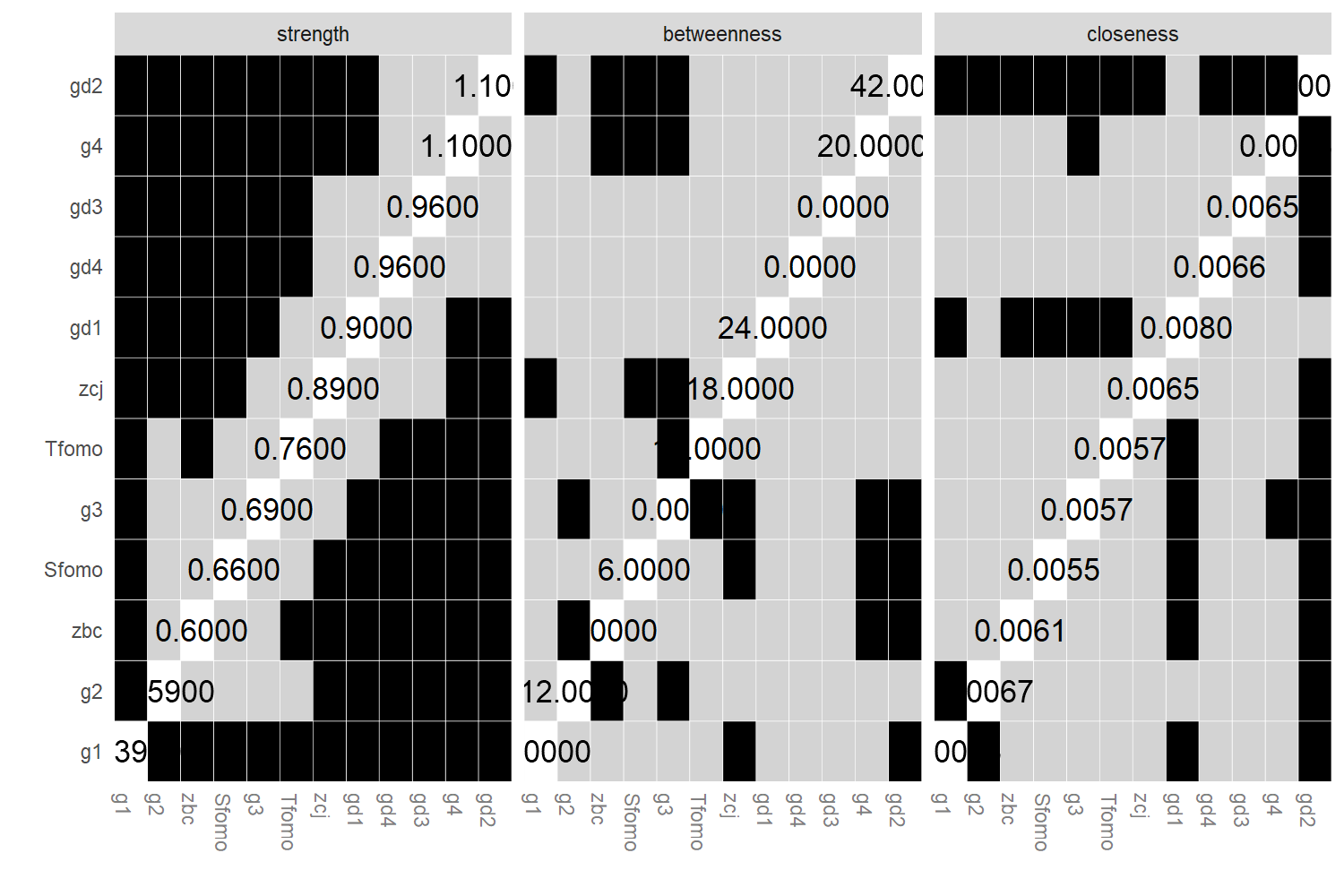

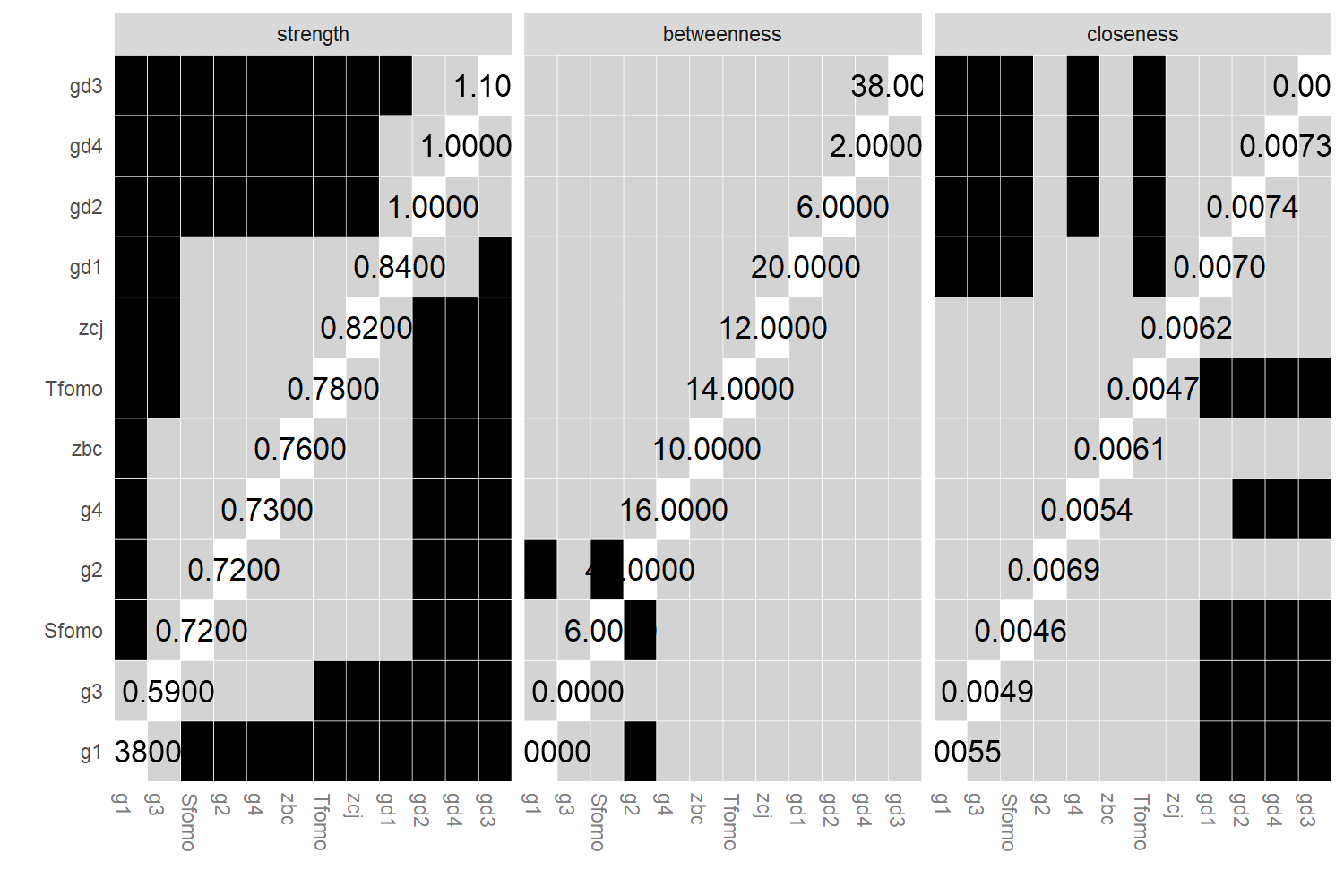


B

A

**Appendix S17.** Centrality stability (parametric) of the facet-level network between 913 males (A) and 722 females (B).

**Appendix S18.** Centrality measures per variable of the item-level network between males and females

| Variable | Males | | | Females | | |  |
| --- | --- | --- | --- | --- | --- | --- | --- |
|  | Betweenness | Closeness | Strength | Betweenness | Closeness | Strength | |
| Bc1 | -0.874 | -0.805 | -0.727 | 0.200 | -0.150 | 0.339 | |
| Bc2 | -0.627 | -0.402 | 0.553 | -0.485 | -0.365 | 0.506 | |
| Bc3 | 0.111 | 0.177 | 1.250 | -0.526 | -0.426 | 0.982 | |
| Cj1 | -0.104 | 0.603 | -0.407 | 0.240 | 0.066 | -0.910 | |
| Cj2 | -0.320 | 0.653 | 0.178 | -0.647 | -0.516 | -4.930e -4 | |
| Cj3 | 0.173 | 1.297 | 0.177 | -0.364 | -0.155 | 0.570 | |
| Cj4 | 2.326 | 2.372 | -0.140 | -0.445 | -0.089 | -0.644 | |
| Cj5 | 2.511 | 2.237 | 0.809 | 0.886 | 0.644 | 0.956 | |
| Fomo1 | -0.658 | -0.261 | 0.430 | -0.808 | -1.284 | **1.371** | |
| Fomo10 | -0.843 | -1.598 | -0.786 | 0.402 | -0.132 | -0.062 | |
| Fomo11 | -0.874 | -1.923 | -0.030 | -0.929 | -1.019 | -0.169 | |
| Fomo12 | -0.135 | -1.157 | -0.147 | -0.888 | -0.860 | -0.234 | |
| Fomo2 | 0.049 | 0.100 | 0.970 | -0.405 | -1.162 | 0.868 | |
| Fomo3 | -0.381 | -0.194 | 0.674 | -0.606 | -0.210 | -0.488 | |
| Fomo4 | 0.603 | 0.182 | 0.899 | 1.692 | 0.627 | 0.489 | |
| Fomo5 | -0.812 | -0.691 | -1.284 | -0.364 | -0.302 | -0.647 | |
| Fomo6 | -0.197 | 0.332 | -0.720 | -0.727 | -0.783 | -0.252 | |
| Fomo7 | -0.504 | -0.581 | 0.541 | -0.526 | 0.102 | 0.469 | |
| Fomo8 | -0.443 | -0.706 | 0.550 | 0.886 | 0.560 | 1.094 | |
| Fomo9 | 2.357 | 0.833 | 0.872 | -0.485 | -0.648 | 0.307 | |
| G1 | -0.874 | -0.948 | -3.100 | -1.090 | -0.823 | -3.132 | |
| G2 | 0.973 | 0.751 | -1.639 | 1.974 | 1.372 | -0.574 | |
| G3 | -0.843 | -0.923 | -1.395 | -1.090 | -1.374 | -2.056 | |
| G4 | -0.104 | -0.502 | 0.825 | -0.042 | -0.609 | -1.140 | |
| Gd1 | -0.874 | -0.072 | -0.357 | 2.176 | 1.850 | -0.185 | |
| Gd2 | 0.849 | 0.546 | **1.377** | -1.090 | 1.501 | 0.546 | |
| Gd3 | -0.874 | 0.120 | 0.271 | 1.813 | 2.190 | 0.952 | |
| Gd4 | 0.388 | 0.562 | 0.357 | 1.248 | 1.994 | 1.044 | |


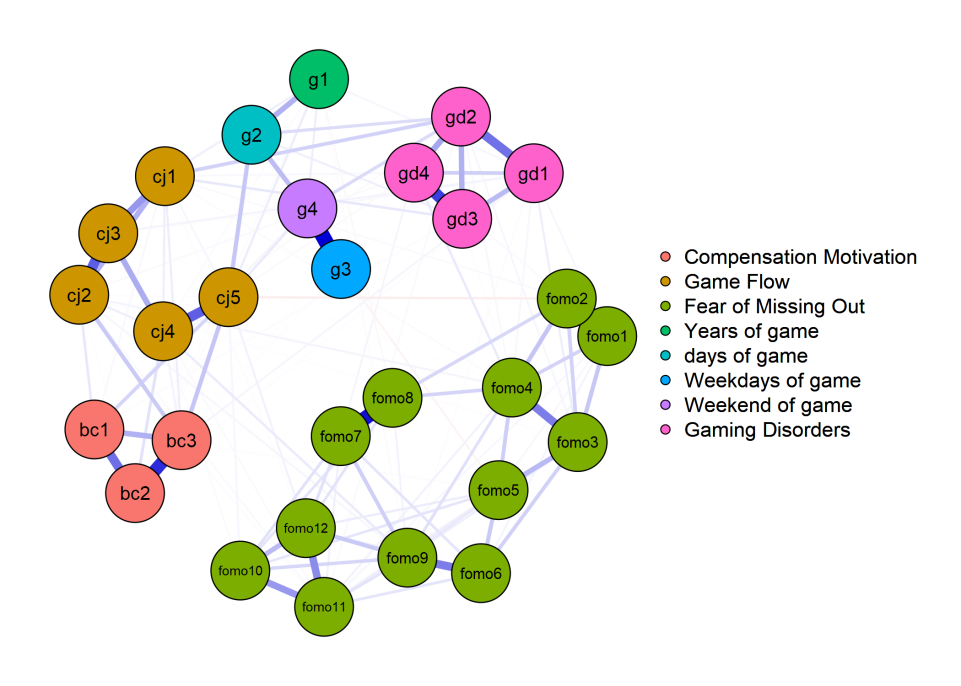

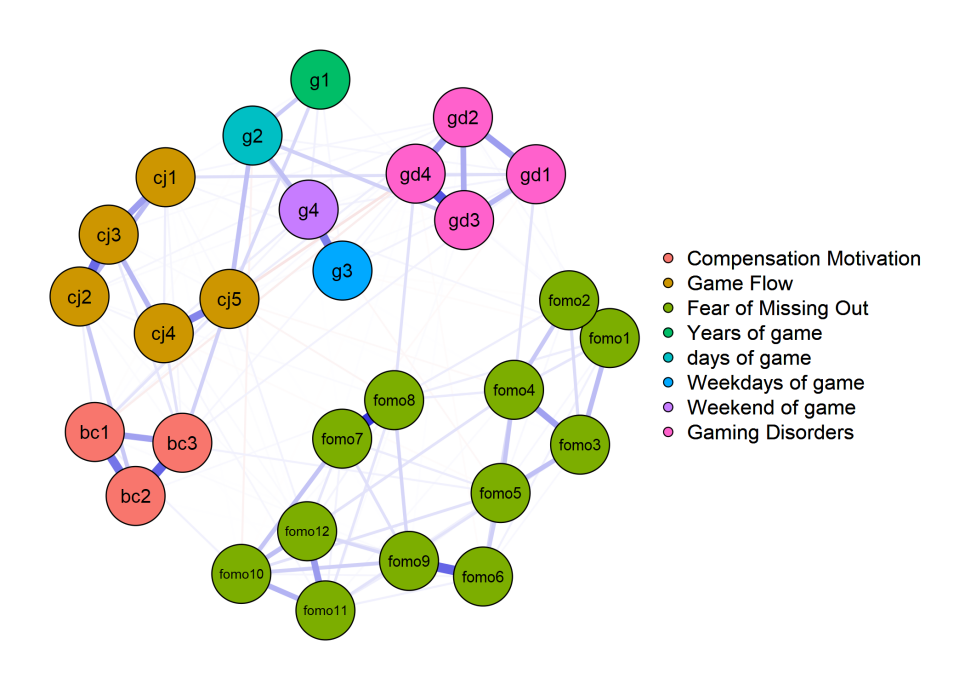


B

A

**Appendix S19.** Item-level EBICglasso model based on network analysis according to the relationships between GD, self-compensation motivation, game flow, FoMO, and game time among 913 males(A) and 722 females (B). Note: gd1～gd4 = Gaming disorder, bc1～bc3 = self-compensation motivation, cj1～cj5 = game flow, fomo1～fomo12 = FoMO, g1～g4= game time.


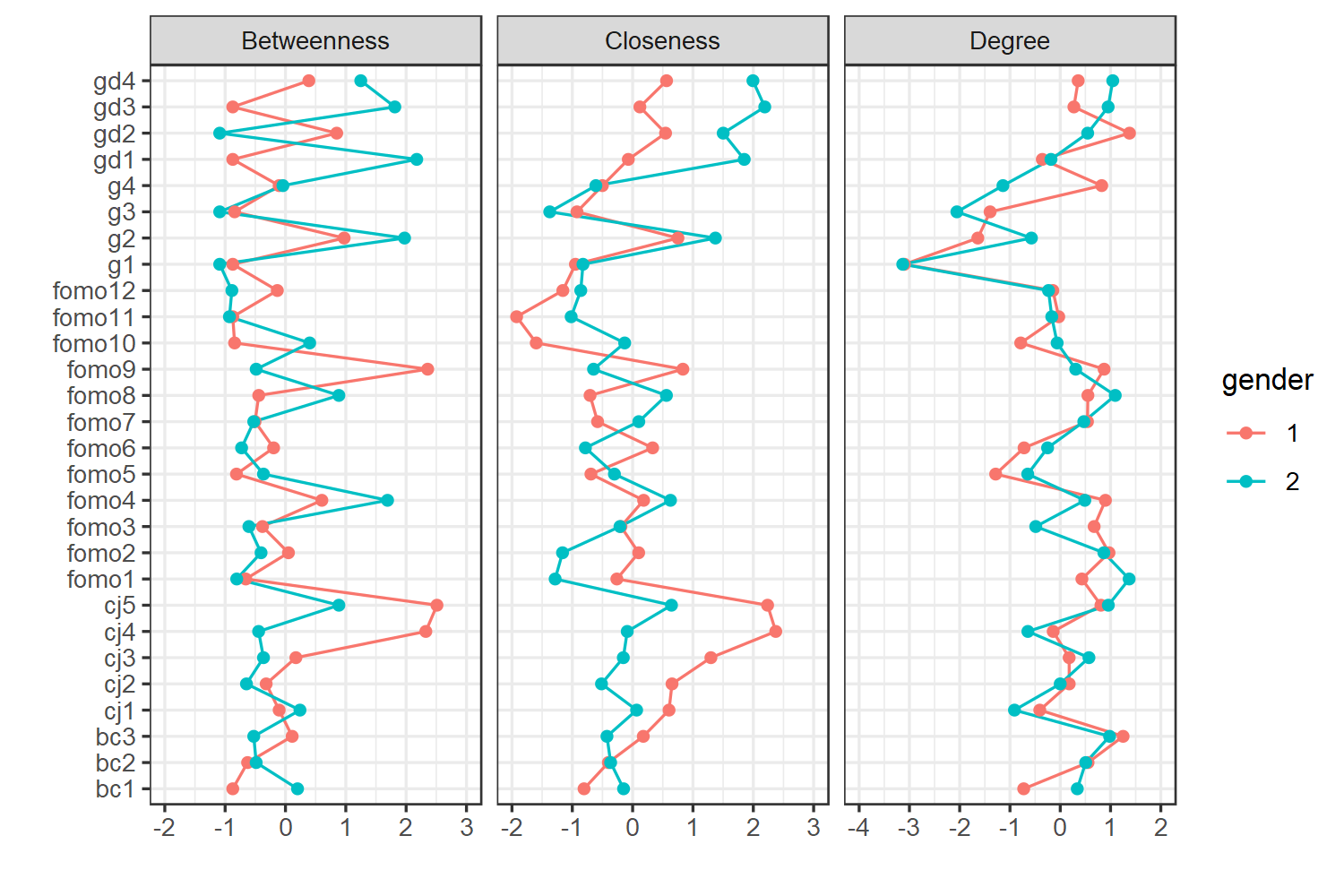


**Appendix S20.** Standardized estimates of node centrality in the item-level network between males and females group. Note: 1 = male, 2 = female.

####
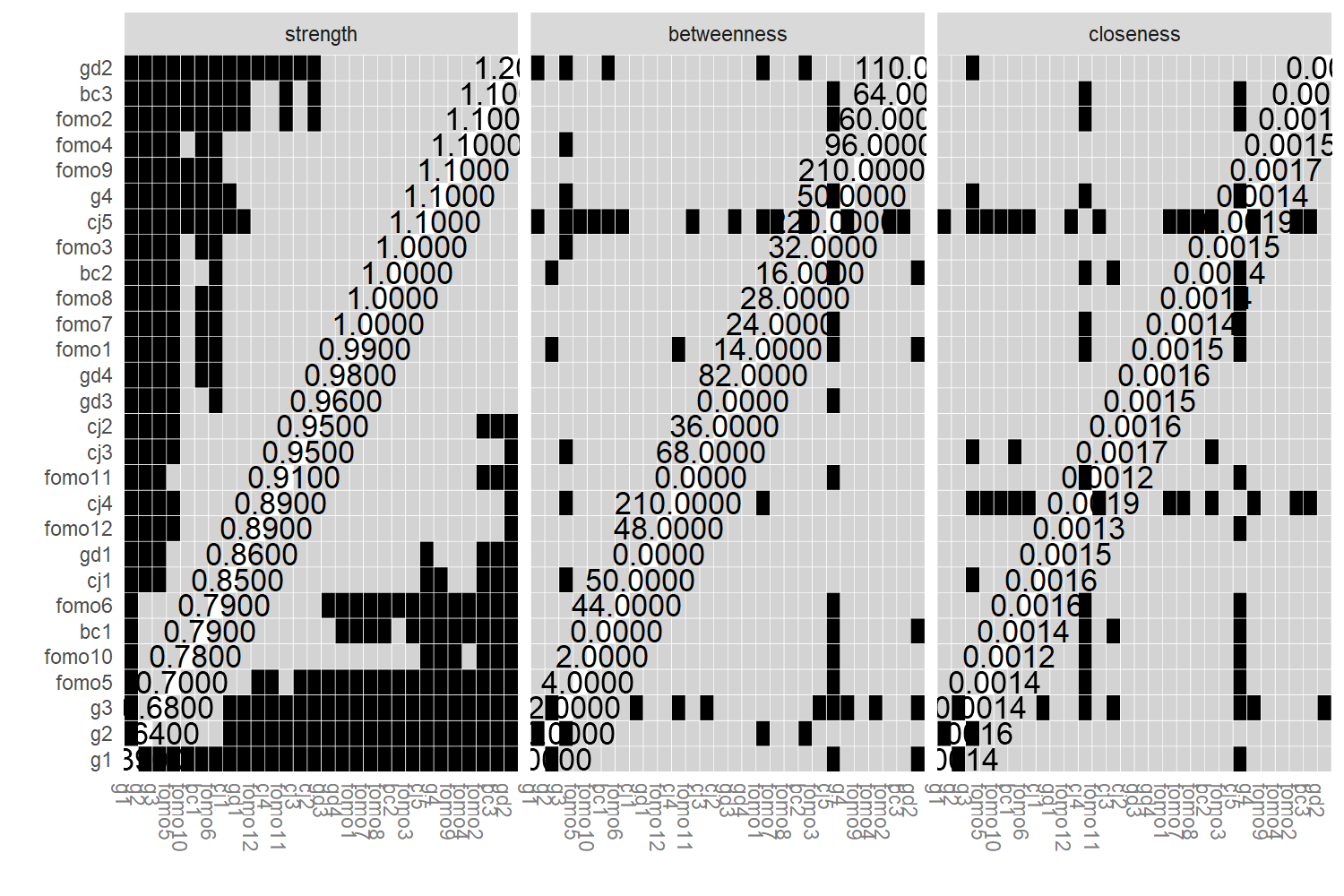

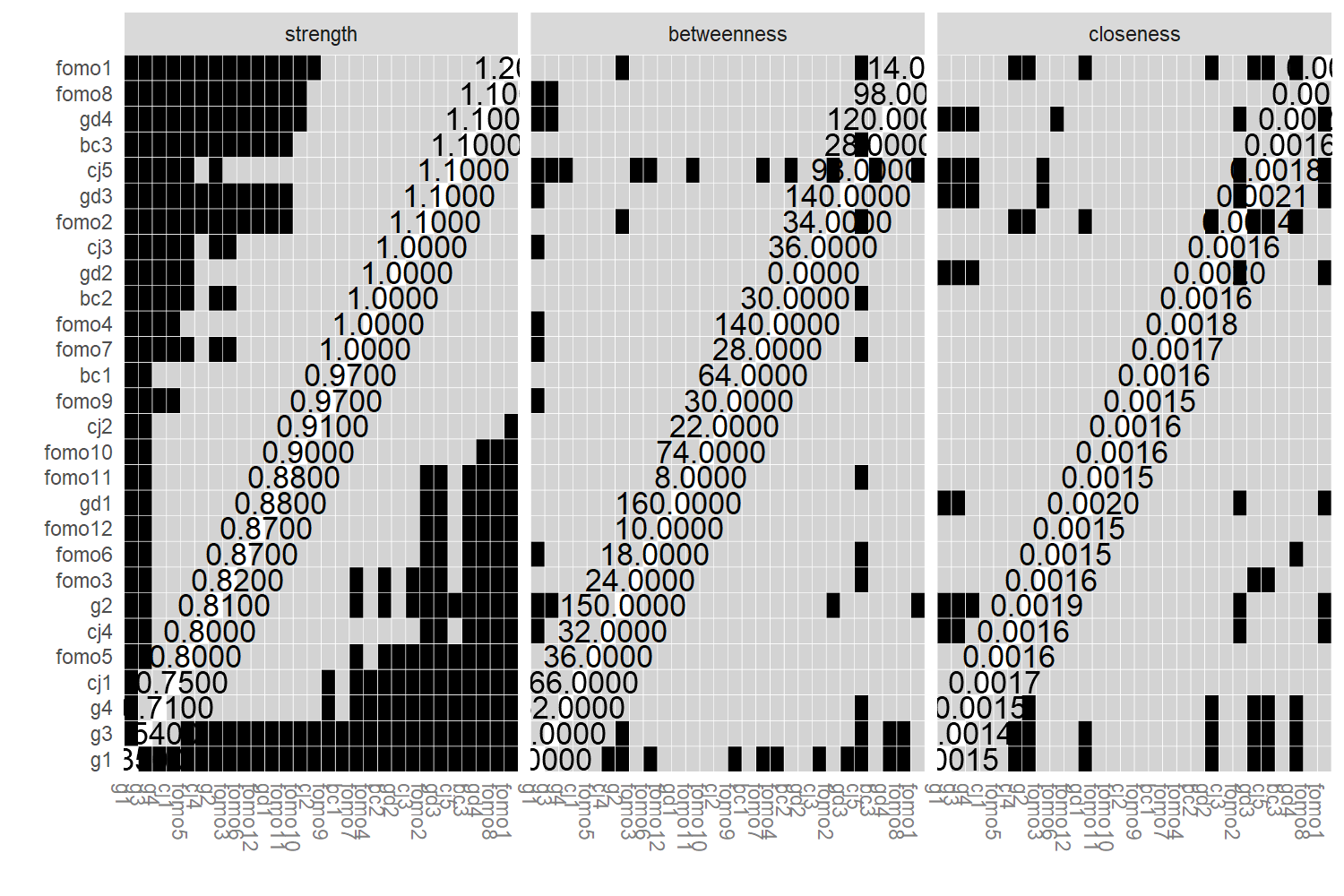


B

A

**Appendix S21.** Centrality stability (parametric) of the item-level network between 913 males (A) and 722 females (B).
